# Gender-based violence and harassment at work and health and occupational outcomes. A systematic review of prospective studies

**DOI:** 10.1186/s12889-024-19304-0

**Published:** 2024-07-04

**Authors:** Katrina J. Blindow, Emma Cedstrand, Devy L. Elling, Malin Hagland, Theo Bodin

**Affiliations:** 1 Institute of Environmental Medicine, Karolinska Institutet, Stockholm, 171 77 Sweden; 2 Center for Occupational and Environmental Medicine, Stockholm Region, Stockholm, 171 77 Sweden; 3 Center for Epidemiology and Community Medicine, Stockholm Region, Stockholm, 171 77 Sweden

**Keywords:** Sexism, Discrimination, Sexual harassment, Sexual assault, Work environment, Adverse social behavior, Mental health

## Abstract

**Background:**

Many people experience forms of gender-based violence and harassment (GBVH) in the context of their work. This includes a wide range of experiences, from subtle expressions of hostility to physical assault, that can also be of a sexual nature (e.g., sexual harassment or assault). This systematic review aimed to summarize findings about the prospective associations of work-related GBVH with people’s health and occupational situation.

**Methods:**

We followed the Preferred Reporting Items for Systematic Reviews and Meta-Analyses guidelines. Scopus, Web of Science, MEDLINE and PsycINFO were searched for prospective studies in English from 1990 to May 24, 2023. Studies were included if they concerned a working population, exposure to any form of GBVH in the work context, and a health outcome or manifest occupational outcome. Quality was assessed with a modified version of the Cochrane ‘Tool to Assess Risk of Bias in Cohort Studies’, and studies assessed as low quality were excluded from the narrative synthesis. For the narrative synthesis, we grouped the results by similar exposures and outcomes and reported the strength and statistical significance of the associations.

**Results:**

Of the 1 937 screened records, 29 studies were included in the narrative synthesis. Studies were mainly conducted in the USA and northern Europe and investigated exposure to sexual violence or harassment (SVH). Only two included studies investigated non-sexual kinds of GBVH. Consistently, studies showed associations of work-related SVH with poor mental health and there were indications of an association with hazardous substance use. There was no consistent evidence for an association of SVH with subsequent sickness absence, and there were too few studies concerning physical health and occupational outcomes to synthesize the results.

**Conclusions:**

There is consistent evidence of work-related SVH as a risk factor for subsequent poor mental health. There is no indication that the health consequences of SVH differ between women and men, although women are more often affected. There is a need for conceptual consistency, the consideration of non-sexual behaviors and prospective studies that test clear hypotheses about the temporal sequence of events.

**Supplementary Information:**

The online version contains supplementary material available at 10.1186/s12889-024-19304-0.

## Background

In 2017, women around the world made some of their experiences of sexism, sexualization and assault known to the public under the joint hashtag #MeToo. A substantial part of these experiences took place in their working lives [1]. Recently, the International Labor Organization (ILO) reported that 8% of women and 5% of men worldwide had experienced sexual violence and harassment (SVH) at work [2]. The ILO acknowledges SVH as a specific kind of gender-based violence and harassment (GBVH), defined as “violence and harassment directed at persons because of their sex or gender or affecting persons of a particular sex or gender disproportionately” [3] and has moved GBVH up the agenda for occupational health and safety [3].

Historically, research of workplace GBVH originated in women’s experiences with the sexual harassment from men [4]. As the research field developed, the scope extended, and the experiences of men and the specific experiences of sexual or gender minorities with workplace GBVH have gained recognition [5]. Today, the field is moving towards an integrated model of GBVH, considering SVH as a form of GBVH, where sexualization is mostly a means of oppression [6–10]. SVH often goes hand in hand with non-sexualizing sexist behaviors [9–12]. Concepts like “gender policing” [7, 13], “gender harassment” [14], “heterosexist harassment” [15], “microaggressions” [16, 17] or “selective incivility” [18], to just name a few, capture demeaning behaviors with differences in content, degree of overt hostility and intent to harm. These conceptual advances have led to a more comprehensive and differentiated understanding of the behaviors people are subjected to, based on their gender and sexuality [8, 13, 15, 18–21]. Here, we align with the definition of the ILO, and consider any interpersonal adverse behavior, that the affected person considered as based on an aspect of their gender or sexual identity as GBVH, including SVH.

Considering the high prevalence of GBVH in some working populations, successful transformations toward workplaces that are safe from GBVH could be an opportunity for gains in population health [21]. Prior reviews of an extensive body of research [5, 22] concluded that GBVH is associated with poor health and reduced occupational well-being [12, 23, 24]. However, these reviews included mostly cross-sectional studies [12, 23–25], which poses challenges for quantifying the population health burden, that can be attributed to the victimization with GBVH. Cross-sectional studies assess exposure and outcome simultaneously and can therefore not determine the temporal sequence of events. This renders it impossible to draw conclusions about the causal relationship between the exposure to GBVH and the health of the affected. Additional to the methodological advantages, prospective studies have the potential to distinguish immediate from delayed effects, further our understanding of the longevity of the impact of GBVH victimization and identify tendencies of health deterioration or recovery. These methodological concerns and limitations of cross-sectional studies are widely acknowledged. However, in most previous reviews, study design or sources of bias that impact study quality were not taken into consideration [12, 23–26]. The two systematic reviews we are aware of, that took study quality into consideration, had a wider scope regarding workplace adversities and concerned a specific occupational [27] or demographic [28] group. They identified very few eligible studies about GBVH.

When GBVH occurs in the context of work, the complex interplay between people’s occupational situation and health may be crucial for the impact of the mistreatment on their health. GBVH can in some cases be a specific form of bullying [29] and has the potential to push people out of their employment [8]. Besides the immediate impact, GBVH may therefore impact the health of the subjected person through career damages, income loss and other work-related factors. Therefore, occupational outcomes are important aspects to consider in the relationship between GBVH and health. The more immediate and versatile occupational attitudes, e.g., job satisfaction or turnover intention have already been synthesized by several meta-analyses [12, 24, 30, 31]. There is, however, no systematic review of the manifest consequences of GBVH on occupational outcomes, such as, e.g., actual turnover or loss in income.

*S*everal contextual factors are potentially relevant for the impact of GBVH. Most prominently, men tend to differ from women in the contexts [32, 33] and the nature of GBVH experiences, as well as their perception of experiences as threatening or harassing [33–37]. Women and men may therefore differ in their vulnerability, and sexual and gender minorities may be particularly vulnerable due to minority stress [26, 38]. Another decisive factor for the health impact of GBVH may be organizational power differentials, e.g., if the abusive behavior stems from a superior, co-worker, or third party [24, 26, 31]. Particularly the difference between harassment experiences from members of the work organization and third parties has not been investigated systematically. They occur, however, in different contexts and may even be of a different nature. Furthermore, while labor laws in many countries hold employers responsible for abuse from co-workers, these laws do not necessarily apply for third party contacts. Also, third-party contacts tend to be brief, while co-workers often constitute a consistent part of each other’s work environment. Furthermore, abusive behavior from customers, clients or patients is highly normalized in some sectors, e.g. in the hospitality industry or health care. Therefore, organizations may also need to take different measures to prevent and respond to GBVH from inside the organization and from third parties.

### Aim

This systematic review aims to assess the evidence of the prospective association of workplace GBVH with the health of the affected. Given the role of their occupational situation as a potential mediating or moderating factor in this association, we also include manifest occupational outcomes. We further assess if the gender of the victimized person and the perpetrator or other contextual factors play a decisive role for the health impact of GBVH.

## Methods

We followed the Preferred Reporting Items for Systematic Reviews and Meta-Analyses (PRISMA) guidelines [39]. The review protocol was registered in PROSPERO (registration number: CRD42023429973). Deviations from the protocol are described in Appendix 1, Additional file 1.

### Search strategy

KJB developed the search strategy in consultation with a librarian at Karolinska Institutet (Stockholm, Sweden), sent it to three external researchers with expertise in relevant fields and added terms based on their suggestions. Searches were conducted on May 24, 2023, in the electronic databases Scopus, Web of Science, Ovid MEDLINE and PsycINFO. Additionally, KJB searched the reference lists of systematic reviews and the eligible studies from the database search. Only studies in English and published from 1990 to the date of the search were considered. The search strings are presented in Appendix 2, Additional file 1.

### Study selection

After deduplication by the librarian, the records were imported to Rayyan QCRI. TB and KJB screened titles and abstracts independently. In case of disagreement, the record was retrieved in full text. KJB and one more author (EC, DLE, or MH) independently assessed the full-text articles. Decisions and motivations were documented in Rayyan. After the independent assessment, decisions were discussed in pairs. Unresolved conflicts were blinded and assessed by TB and resolved in consensus. Ambiguous cases were collected and used to systematically discuss our inclusion criteria and – where necessary – specify them.

### Eligibility criteria

#### Setting and participants

Studies were considered eligible if they (i) included individuals of working age (15 to 68), (ii) who participated in the labor market, employed or self-employed (including interns, apprentices, and doctoral students when they are exposed to a work environment rather than an educational setting). We focus on the formalized, legal labor market. Therefore, studies about individuals in informal and illegal work contexts were not included. For sex work, this implies that the inclusion depended on the country legislation.

#### Exposures

Studies were eligible if they assessed experiences that classify as GBVH. This includes any kind of incivility, violence, harassment from a specific person with gender discriminating content or which the affected person ascribed to an aspect of their gender identity (including sexual identity). Experiences of sexual harassment and assault were included, regardless if the affected person regarded them as gender-based. All definitions of sexual harassment by researchers were accepted. Witnessing the harassment of others, general assessments of the workplace culture, discrimination that is not clearly attributable to a person, i.e., in hiring, promotion, or pay, or assessments of discrimination or harassment where gender was one of many possible grounds (e.g., alongside ethnicity or age) were not included. Only studies where the exposure clearly occurred in the work context were eligible.

#### Comparators

Studies were eligible if exposed individuals were compared to none or less exposed individuals from the same population.

#### Outcomes

Any health outcome, self-reported or from other sources, as well as sickness absence and treatment (seeking) were included. Further, manifest occupational outcomes (e.g., turnover) were included, but not measures of attitudes (e.g., satisfaction) or intent (e.g., turnover intention).

#### Study design

Only studies with a prospective design were eligible, meaning that the exposure was assessed before the outcome. We further included only studies where the main potential confounders age and gender were taken into consideration.

### Data extraction

Preliminary extraction of relevant information was performed independently by KJB and one more author (EC, DLE, or MH) during quality assessment, into a google form. Corresponding authors of the articles were continuously requested to provide missing information or resolve ambiguities by email. The final data extraction was conducted by KJB in consultation with TB. The following information was extracted: authors, year of publication, country, population characteristics (e.g., occupation/industry) and exclusion criteria, sample size, age and gender composition, exposures (constructs and operationalizations), percent exposed, outcomes (constructs and operationalizations), percent cases, co-variates, time lag/follow-up time, statistical method, risk estimate, gender differences in the association.

If results indicated differences between women and men in the association, stratified results were extracted. Gender-stratified results were not extracted if results from the full sample were available and no interaction with gender was found. When results for binary outcomes were presented, results from linear regression for related outcomes were not extracted. When relevant composite outcomes were reported, results for the individual outcomes, that are included in the composite measure, were not extracted.

### Quality assessment

The risk of bias of studies was assessed independently by KJB and another author (EC, DLE, or MH). The assessments were then discussed in the respective pairs, who agreed on a final score. An exception was made for the two studies that are authored by KJB, they were assessed by EC and MH. We applied a modified version of the ‘Tool to Assess Risk of Bias in Cohort Studies’ (see methods.cochrane.org) in the google form (see Appendix 3, Additional file 1). We added risks of bias with a total score of 0–29 points. A lower score indicates better quality. Sample representativeness could be rated as 0 or 1, all other dimensions as 0–3. We rated the risk of bias regarding the assessments of exposures, outcomes, and confounders, adjustments for relevant confounders or consideration of outcome status before exposure, follow-up time, or loss at follow-up. We further assessed if adequate statistical methods were used and provided a rating for “miscellaneous”, where an unanticipated weakness could be added with a comment and be rated. We considered ≤ 5 points as high, 6–9 points as medium and > 9 as low quality. We also applied an additional rule, articles that have at least one dimension rated as high risk of bias could not be considered high quality. These articles were therefore downrated to moderate quality regardless of the total score.

### Synthesis of study results

Only studies with moderate or high quality were included in the synthesis of results. Due to the high diversity in outcomes, study designs and types of effect estimates, conducting a meta-analysis was not appropriate. The results are presented in a narrative synthesis, following the ‘Synthesis Without Meta-analysis (SWiM) reporting guideline’ [40]. When several studies were conducted on the same cohort, investigating the same or similar exposure-outcome-associations, we only considered one study for synthesis. We prioritized studies with higher quality ratings, reporting interpretable risk estimates, or results from validated scales.

We used tabulation to group studies by exposure and the investigated outcomes and counted if the estimates of association were similar in statistical significance, direction, and strength. When estimates of relative risk (i.e., odds ratios, risk ratios or hazard ratios) were presented, the strength of the association was divided into the three categories weak (1.01–1.20) moderate (1.21-2.00) and strong (> 2.00). When coefficients from linear regression were presented, we interpreted the strength of the association in consideration of the included scales. When articles only presented results for women and men separately, we considered them as two different samples. When results were also presented for the whole sample, we only included these.

We considered the evidence for a prospective association between an exposure and outcome to be consistent when measures of effect were mostly in the same direction, similar in strength and statistically significant in analyses that had sufficient power to find a true effect of moderate strength.

We conducted additional syntheses to explore if the associations between exposures and health outcomes differed depending on contextual factors, including all health outcomes. First, we investigated gender differences in the associations. Second, we sorted results regarding the definition of harasser characteristics and compared associations. Third, we compared the strength of the associations of GBVH with health outcomes between different methods of exposure assessment.

## Results

**Fig. 1:**
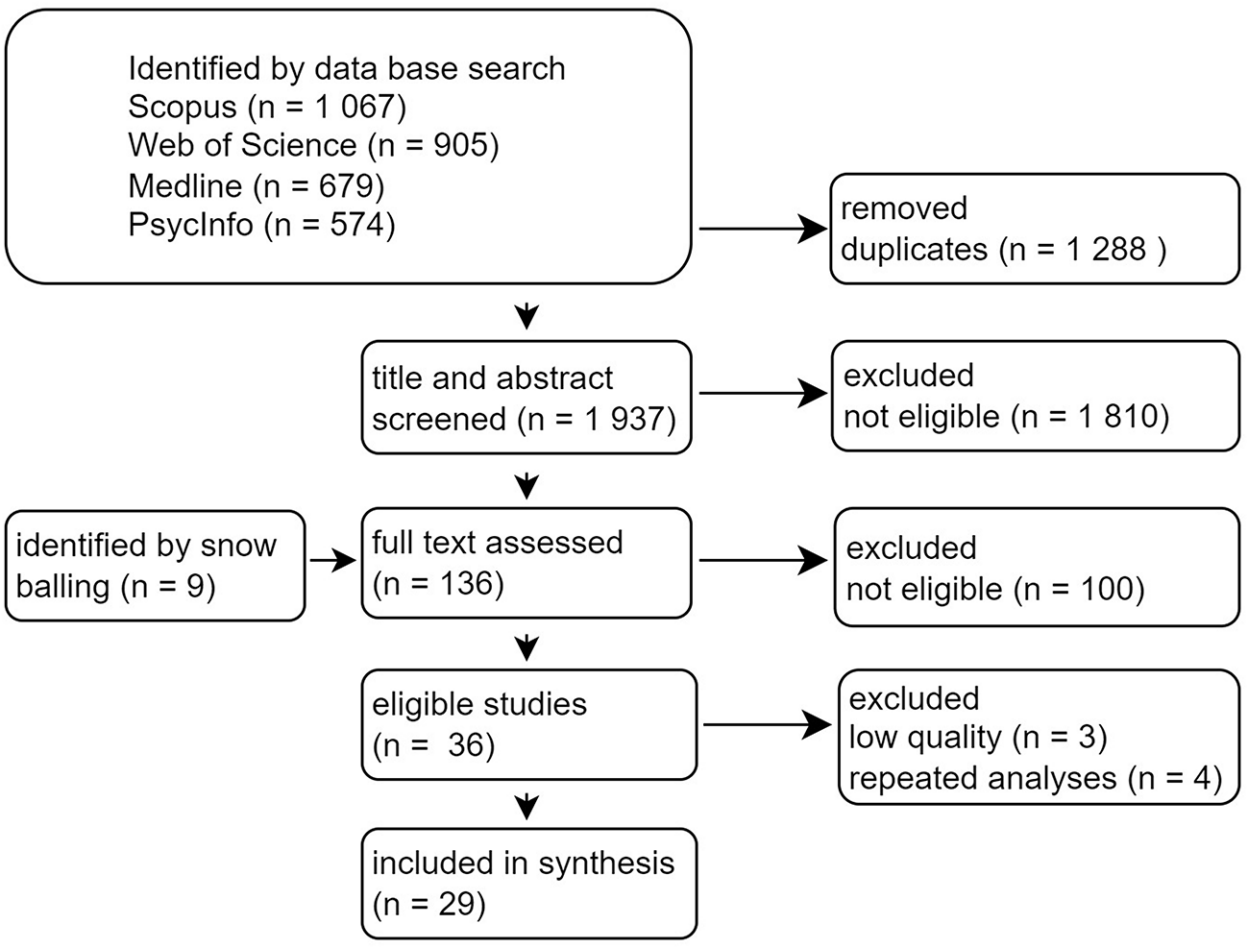
Flow diagram of study selection

An overview of the selection process is presented in Fig. 1. A total of 3 225 records were identified by database searches. After de-duplication, 1 937 records entered abstract and title screening. Of those records, 127 were selected for full-text screening, and nine records were added from searching reference lists or prior knowledge of the literature. Of the 136 articles that were retrieved in full-text, 100 were assessed as not eligible, primarily because the study design or exposure was out of scope (see Appendix 4, Additional File 1 for excluded studies and reasons for exclusion). This resulted in 36 eligible articles. We further excluded three studies from synthesis due to low quality [41–43], and five studies reported results from similar analyses based on the same cohort. This led to further exclusion of four studies [44–47] (see Appendix 5, Additional file 1 for details). A total of 29 studies were included in the narrative synthesis.

Among the excluded studies, several may appear to meet the inclusion criteria. We were open to the inclusion of studies, where the term “discrimination” clearly referred to interpersonal behaviors. In one study, a survey item assessed “gender discrimination”, but did not specify interpersonal behavior and could be interpreted to aim at hiring, promotion or wage discrimination [48]. We excluded this study. We also excluded what we call “onset studies” [49, 50]. In this study design, two survey waves are used, and the invested association concerns the health outcome in relation to the onset of exposure, determined by the absence of exposure in the previous wave. A special case was one study, that included only individuals who reported sexual harassment at baseline and investigated the association of continuing versus “remission from” harassment with the health status at the second survey wave [51]. While we acknowledge that these are interesting study designs, we excluded the studies on the grounds that they are not prospective.

We sorted studies by cohort (ordered by first study published) and, within cohorts, by date of publication (see Appendix 6, Additional file 1). The included 29 studies were published between 2000 and 2023. We included 15 studies from the USA, 13 studies from northern Europe, and one study from China. They were conducted on 18 different samples, of which some consisted of several pooled cohorts. The mean age of the included samples ranged from 19 to 53 years.

Most studies focused on SVH, specifically unwanted sexual attention [29, 52–56], sexual harassment [32, 37, 57–70], severe sexual harassment [71] or Military Sexual Trauma (sexual harassment or assault during military service) [72–75] with differing constructs and operationalizations. We synthesized therefore only the results concerning prospective associations of SVH and different health and occupational outcomes. Only two studies also investigated non-sexual GBVH [57, 58], those results can be found in Appendix 6, Additional file 1.

In most studies, exposure was assessed with a survey. In the USA, exposure was commonly assessed with the behavior-based approach. Participants were presented with a list of potentially harassing experiences, and varying cut-offs were applied to determine cases. In the USA, predominantly, a version of the Sexual Experiences Questionnaire (SEQ) was used. In one Norwegian study, the Bergen Sexual Harassment Scale (BSHS) was used [76]. In most other studies from northern Europe, sexual harassment or unwanted sexual attention was assessed with the self-labelling approach, i.e. a direct question about exposure. Recall time was not always reported [54–56, 70]. When it was stated, it was the past 12 months or the last year [32, 52, 53, 57, 58, 60, 62, 64–69, 71, 77, 78], 6 months [37, 53], during lifetime [61, 63], or during recruit training [59]. In four studies from the USA, information about Military Sexual Trauma was gained from health care records [72–75].

**Table 1:** Results from included studies of the prospective associations of sexual violence and harassment with mental health outcomes, physical health outcomes, substance use (disorder) and sickness absence, including gender differences in the exposure-outcome association (♀♂) and quality assessment (Q)

Reference	Sample	*N* ^a^ (% men)	Time-lag Follow-up	Exposure^b^	Outcome^c^	Effect size (CI or SE)^d^	♀♂ ^e^	Q^f^
***Physical health outcomes***								
Gaffey et al., 2022	Military service veterans, USA	788 161 (87)	Mean follow-up 10 years	Military sexual trauma (harassment or assault); VHA screen; two items	Incident hypertension; diagnosis or treatment for hypertension; register-based	HR 1.15 (1.11–1.19)	♀+	M 6
Lawn et al., 2022	Female nurses, USA	33 127 (0)	Biannual follow-up 2008–2015	Lifetime sexual harassment at work; one item, specifying physical and verbal harassment; No trauma / other (non-sexual harassment) trauma / sexual harassment	Incidence hypertension; self-reported high blood pressure, diagnosis or treatment	HR 1.12 (1.03‒1.22)	♀♀	M 7
***Mental health outcomes***								
Blindow et al., 2022	National sample, SE	22 467 (≈ 49.1)	Mean follow-up 6.4 years	Sexual harassment by a superior, colleague or third party past 12 months; one item	Incidence dispense of psychotropic medication; register-based	Once: HR 1.15 (0.99 to 1.33) Mo-daily: HR 1.37 (1.12 to 1.67)	♀=♂	H 4
Brignone et al., 2017	Military service veterans, USA	485 884 (88)	5-year continuous follow-up	Military sexual trauma (harassment or assault); VHA screen; two items	Use of outpatient mental health care, register-based	OR 2.82 (2.62–3.05)	♂+	M 9
					Use of inpatient psychiatric care, register-based	OR 2.57 (2.30–2.87)	♂+	M 9
Gradus et al., 2013	Members of recruit training for the Marines, USA	646 (≈ 46)	10-year time-lag	Sexual harassment during recruit training; SEQ	Attempted Suicide; one-item and register	OR 2.8 (1.2–6.6)	n.t.	M 8
Gross & Ronzitti et al., 2020	Female Veterans of military service, USA	750 176 (88)	follow-up n.r.	Military sexual trauma (harassment or assault); VHA screen; two items	Nonfatal Severe Self-Directed Violence (SDV) resulting in inpatient hospitalization; ICD codes; register-based	Male with MST: HR 1.28 (1.10–1.48) Female without MST: HR 1.05 (0.94–1.18) Female with MST: HR 1.63 (1.46–1.83)	♀+	M 9
Houle et al., 2011	Cohort of public school 9th -graders, age 30–31 at study baseline, USA	732 (42)	1-year time-lag	Sexual harassment by a supervisor, co-worker, customer, or client past year, ISH and SEQ	Depressive affect; 4 items from GWS; continuous scale	B coef 0.217 (SE 0.062) *p* < 0.001	♀=♂	H 5
Magnusson Hanson et al., 2020	National sample, SE	82 860 (≈ 48)	Mean follow-up 13 years	Sexual harassment by a superior, colleague or third party past 12 months; one item	Suicide, register-based	HR 2.47 (1.25–4.87)	♀=♂	H 4
		82 233 (≈ 48)			Suicide attempt, register-based	HR 1.56 (1.18–2.05)	♀=♂	H 4
Nielsen et al., 2012	National sample, NO	Women: 976 Men: 799	2-year time-lag	Sexual harassment at present workplace or work-related social event last 6 month; BSHS	Psychological distress; HSCL-25; <1.75/≥1.75	Women: OR 2.03 (1.2–3.39) Men: OR 1.32 (0.72–2.43)	♀+	H 4
Rospenda et al., 2006	University employees, USA	≈ 1 368 (44)	3-year time-lag	Sexual harassment in the work setting last year; SEQ	Services use past 3 years to deal with work-stress; one item; battery of health care or non-health professionals as response options	Remission: OR 1.57 (1.06–2.32) Intermittent: OR 2.87 (1.24–6.65) Chronic: OR 2.56 (1.75–3.75)	♀=♂	M 8
Rugulies et al., 2020	National sample, DK	6 647 (47)	2-year time-lag	Sexual harassment last 12 months; one item; No exposure/exposure by non-workplace personnel (non-WP)/exposure by workplace personnel (WP)	Depressive symptoms last 2 weeks; MDI	Non-WP: b coef 0.76 (-0.65–2.18) WP: b cof. 2.54 (0.62–4.46)	♀=♂	M 4^**g**^
Sterud, Hanvold et al., 2021	National sample, NO	3654 (51)	≈ 3-year time-lag	Unwanted sexual attention at the workplace; one item	Mental distress last 2 weeks; HSCL-5; <2.0/≥2.0	OR 1.64 (1.03 − 2.61)	♂+	H 4
Shannon et al., 2007	National sample, USA	1 196 (≈ 53)	1-year time-lag	Sexual harassment at the job past 12 months; modified SEQ	Service use past 12 months to deal with work-stress	Chronic: OR 1.45 (0.94–2.23) Remission: OR 1.16 (0.72–1.89)	n.t.	M 6
Wolff et al., 2017	First-year university students who also worked, USA	Women: 925 Men: 640	≈ 6 months time-lag	Sexual harassment from bosses, co-workers, or customers/clients past 12 months; 13 items from SEQ	Depressive symptoms past week, seven items from CESD, count	Women: 0.03 (SE 0.01) *p* < 0.05 Men: 0.04 (SE 0.03) *p* > 0.05	♀=♂	M 5
Zhu et al., 2018	Service workers in hotels, CN	266 (20)	1-month time-lag	Sexual harassment by supervisors/coworkers/customers (time not specified); SEQ	Depression past week; CESD, 20 items; continuous scale	B coef. 0.21 (*p* < 0.01)	n.t.	M 8
***Substance use (disorder)***								
Brignone et al., 2017	Military service veterans, USA	485 884 (88)	5-year continuous follow-up	Military sexual trauma (harassment or assault); VHA screen; two items	Use of outpatient substance use care over 5-year period; register-based	OR 2.12 (1.91–2.36)	♀=♂	M 9
					Use of inpatient substance use care over 5-year period; register-based	OR 1.73 (1.22–2.44)	♀=♂	M 9
Goldberg et al., 2019	Military service veterans, USA	435 690 (≈ 87)	≥ 5 years continuous follow-up	Military sexual trauma (harassment or assault); VHA screen; two items	Drug use disorder; ICD codes, register-based	OR 2.26 (2.09–2.43)	♀+	M 9
		390 833 (≈ 87)			Alcohol use disorder; ICD codes, register-based	OR 1.63 (1.49–1.79)	♀+	M 9
Rospenda et al., 2008	National sample of employed residents, USA	Women: 722 Men: 733	1-year time-lag	Sexual harassment at the job past 12 months; modified SEQ, 9 items	Frequency of heavy episodic drinking 5 + drinks on the same occasion; one item; count	Women: IRR 1.13 (0.93–1.37) Men: IRR 1.41 (1.10–1.57)	n.t.	M 7
Wislar et al., 2002	University employees, USA	1 433 (≈ 47)	1-year time-lag	Sexual harassment in the work setting last year; 19 items from modified SEQ,	Problem drinking past 12 months; MAST score; <4/≥4	remission: OR 1.46 (0.94–2.26) chronicity: OR 1.35 (0.91–2.01)	n.t.	M 6
Wolff et al., 2017	First-year university students, USA	Women: 926 Men: 640	≈ 6-months time-lag	Sexual harassment from bosses, co-workers, or customers/clients past 12 months; 13 items from SEQ	Alcohol-related problems, RAPI score, count	Women: b coef logit: 0.14 (SE 0.10) *p* > 0.05 b coef count: 0.07 (SE 0.02) *p* < 0.01 Men: b coef logit: -0.13 (SE 0.25) *p* > 0.05 b coef. count: 0.09 (SE 0.04) *p* < 0.05	♀=♂	M 5
***Sickness absence***								
Blindow et al., 2021	National sample, SE	Women: 28 998 Men: 27 588	1-year follow-up	Sexual harassment by a superior or colleague past 12 months, one item with definition	Long-term sickness absence; <21/≥21 consecutive days, register-based	Women: Once: RR 0.99 (0.97–1.02) Mo-daily: RR 1.06 (1.01–1.10) Men: Once: RR 1.02 (1.00–1.03), Mo-daily: RR 1.04 (1.02–1.05)	♀=♂	H 5
Clausen et al., 2012	Elder care employees, DK	9520 (0)	1-year continuous follow-up	Unwanted sexual attention at work past 12 months; one item	Sickness absence ≥ 8 consecutive weeks; register-based	Occasional: HR 0.99 (0.74–1.32) Frequent: HR 1.46 (0.75–2.82)	♀♀	H 5
Hogh et al., 2016	National samples; DK	Women: 9 599 Men: 9 767	18-months continuous follow-up	Unwanted sexual attention from colleagues, managers and/or subordinates past 12 months, one item	Incidence long-term sickness absence; <15/≥15 consecutive days, register-based	Women: HR 1.10 (0.60-2.00) Men: HR 2.47 (1.32–4.65)	♂+	H 5
				Unwanted sexual attention from clients/customers/patients past 12 months, one item		Women: HR 0.89 (0.52–1.51) Men: HR 1.31 (0.67–2.54)	♀=♂	H 5
Nabe-Nielsen et al., 2016	National sample and hospital and civil-service employees, DK	8 669 observa-tions (≈ 30)	2-year continuous follow-up	Unwanted sexual attention at work past 6 or 12 months; one item	Long-term sickness absence, register-based < 30/≥30 consecutive days	OR 1.61 (1.11–2. 41)	♀=♂	H 4
Sterud, Degerud et al., 2021	National sample, NO	LLSL: 18 179 HLSL: 17 685 Observations (≈ 52)	1-year continuous follow-up	Unwanted sexual attention at the workplace; one item	Cumulative sick leave days/calendar year; register-based; 0/1–16 (low level, (LLSL))/>16 (high level (LSL)	LLSL: OR 1.35 (1.09 to 1.67) HLSL: OR 1.41 (1.10 to 1.79)	♀=♂	H 4

^a^Number of individuals in the analytical sample. Where the exact number for the analytical sample was not retrievable (not reported and not delivered on request), percentages as reported for the whole study sample are presented if available. This is indicated by the symbol ≈

^b^Instruments for exposure assessment: ISH = Inventory of Sexual Harassment, SEQ = Sexual harassment questionnaire, BSH = The Bergen Sexual Harassment Scale, VHA screen: Veterans Health Administration screening instrument, NAQ = Negative Acts Questionnaire

^c^Instruments for outcome assessment: MDI = Major Depressive Inventory; CESD = Center for Epidemiologic Studies Depression Scale; BJSQ = Brief Job Stress Questionnaire; RAPI = Rutgers Alcohol Problems Index, ICD = International Statistical Classification of Diseases and Related Health Problems; EPDS = The Edinburgh Postnatal Depression Scale; GWS = General Well-being Scale of the Current Health Insurance Study Mental Health Battery; HSCL = Hopkins Symptoms Checklist

^d^If nothing else is stated, numbers in brackets are the 95% confidence interval; SE = standard error, OR = odds ratio, HR = hazard ratio, IRR = incidence rate ratio, RR = risk ratio, ATET = average treatment effect of the treated

^e^Difference in the association between the exposure and outcome among women versus among men; n.t. = not tested; ♀=♂ = no gender difference found; ♀+ = stronger association in women ♂+ = stronger association in men; ♀♀ = only women in the sample; ♂♂ = only men in the sample

^f^Quality assessment and total score; M = Medium quality (6-9); H = High quality (0–5)

^g^Downrated to moderate quality due to high risk of bias due to attrition at follow-up

### SVH and physical health

Two medium quality studies investigated SVH as a risk factor for hypertension. In one study, sexual harassment or assault during military service and hypertension diagnoses were identified in the veteran health care registers [73]. In the other study, sexual harassment and hypertension diagnosis or treatment were assessed with surveys among female nurses [61]. Both studies found a weak, statistically significant association.

### SVH and mental health

In total, 13 studies investigated SVH in relation to prospective poor mental health (see Table 1). The studies were conducted on 12 different samples. The six studies from the USA were conducted on a national sample, university employees, military recruits and veterans. The other studies were conducted on national samples from Sweden, Norway and Denmark, and hotel employees in China. Eight studies were assessed as medium quality and five studies as high quality.

Nine studies were entirely survey-based. In four of these studies, the outcome was depressive symptoms, assessed with validated scales [60, 66, 69, 70]. These four studies found in total six weak associations of sexual harassment with depressive symptoms, of which four were statistically significant. In two studies, psychological distress was determined with a validated scale, and the applied cut-off indicated a need for treatment [54, 76]. These two studies showed two moderate and one strong associations, of which two were statistically significant. Two studies investigated use of services to deal with work stress in relation to prior sexual harassment and showed a weak to moderate but statistically non-significant [77] and one strong association [65].

In three studies, survey responses about sexual harassment were combined with register data. One study found a moderate association with psychotropics use [58] and one study found strong associations with suicide attempts and suicide [62]. One study showed a strong association of sexual harassment during military recruit training with suicide attempts (assessed by survey and health registers) [59]. Two studies used health registers to determine Military Sexual Trauma. One study found a strong association with mental health care use [72], and one study a moderate association with nonfatal severe self-directed violence [75].

In summary, there is consistent evidence for a prospective association between work-related SVH and mental health. Further, it appears that studies with more severe mental health outcomes tend to report stronger associations. Studies with continuous outcomes of depressive symptoms showed weak associations. Studies using cut-offs for treatment-relevant symptom levels and register-based outcomes of mental health treatment, self-directed violence and suicide (attempts) showed mostly moderate or strong associations. This could, however, also be due to differences in study designs.

### SVH and substance use (disorder)

Five included studies investigated substance use in relation to prior SVH (see Table 2). They were all assessed as medium quality. In three studies, alcohol consumption was assessed with self-reports. The studies were all conducted in the USA, on a national sample, (working) university students, and university employees, respectively. Two studies used validated scales to assess hazardous alcohol consumption [68, 69] and one study assessed frequency of heavy episodic drinking [64]. These three studies showed three statistically significant associations (two very weak, one moderate) and three statistically non-significant associations (one weak and two moderate).

**Table 2:** Results from included studies of the prospective associations of sexual violence and harassment with occupational outcomes, including gender differences in the exposure-outcome association (♀♂) and quality assessment (Q)

Reference	Sample	*N* ^a^ (% men)	Time-lag Follow-up	Exposure^b^	Outcome	Effect size (CI or SE)^c^	♀♂ ^d^	Q^e^
Clausen et al., 2013	Elder care, DK	4826 (≈ 2)	≈ 1–1 ½ -year time-lag	Unwanted sexual attention at work past 12 months; one item, no definition	Turnover, register-based and survey item	Occasional: OR 1.33 (1.03–1.71) Frequent: OR 1.06 (0.58–1.93)	n.t.	M 7
Folke et al., 2022	National sample, SE	Women:17 971 Men: 15 486	3-year follow-up	Sexual harassment by a superior or colleague past 12 months; two items with definitions of sexist and sexual hostility and unwanted sexual attention, no/yes	Turnover within 3 years, register-based	Women: ATET 4.15% *P*= 0.000 Men: ATET 3.54% *P* = 0.052	n.t.	H 4
McLaughlin et al., 2017	Public school 9th-graders, followed to age 31, USA	364 (0)	2-year time-lag	Severe sexual harassment supervisor, co-worker, customer, or client past year, meeting legal definitions of hostile work environment; 7 items from ISH and SEQ	Financial stress past year; one item; 7-point scale; continuous	b coef. 0.72 (SE 0.32) *p* ≤ 0.05	♀♀	H 4
Raj et al., 2020		494 (0)	≈ 7-year follow-up	Sexual harassment by a superior or colleague over life-time, one item	Full professor; survey item and public databases	OR 1.25 (0.80–1.95)	♀♀	M 8
		561 (0)			Retention in academics; survey item and public databases	OR 0.69 (0.43–1.10)	♀♀	M 8
		494 (0)		Severe sexual harassment by a superior or colleague over life-time, several items	Full professor; survey item and public databases	OR 1.77 (1.10–2.87)	♀♀	M 8
		561 (0)			Retention in academics; survey item and public databases	OR 0.93 (0.56–1.54)	♀♀	M 8
Sims et al., 2005	Active members of military service, USA	11 521 (0)	4-year follow up	Sexual harassment by supervisors or coworkers past year; SEQ–DoD	Turnover (leaving the military); administrative records	HR: 1.09 (n.r.) *p* < 0.01	♀♀	H 5
Sterud et al., 2023	National sample, NO	17 110 observations (51)	≈ 3-year time-lag	Unwanted sexual attention at the workplace; one item	Health-related employment exit; one item	OR 2.15 (1.36–3.40)	n.t.	H 4

^a^Number of individuals in the analytical sample. Where the exact number for the analytical sample was not retrievable (not reported and not delivered on request), percentages as reported for the whole study sample are presented if available. This is indicated by the symbol ≈

^b^Instruments for exposure assessment: ISH = Inventory of Sexual Harassment, SEQ = Sexual harassment questionnaire, BSH = The Bergen Sexual Harassment Scale, VHA screen: Veterans Health Administration screening instrument, NAQ = Negative Acts Questionnaire

^c^If nothing else is stated, numbers in brackets are the 95% confidence interval; SE = standard error, OR = odds ratio, HR = hazard ratio, IRR = incidence rate ratio, RR = risk ratio, ATET = average treatment effect of the treated

^d^Difference in the association between the exposure and outcome among women versus among men; n.t. = not tested; ♀=♂ = no gender difference found; ♀+ = stronger association in women ♂+ = stronger association in men; ♀♀ = only women in the sample; ♂♂ = only men in the sample

^e^Quality assessment and total score; M = Medium quality (6-9); H = High quality (0–5)

^**f**^Downrated to moderate quality due to high risk of bias due to attrition at follow-up

Two moderate quality studies used health registers of the same cohort of veterans to determine Military Sexual Trauma. One study showed a strong association with drug use disorder and a moderate association with alcohol use disorder [74]. One study showed a moderate to strong association with substance use care [72]. All findings were statistically significant.

In summary, there are indications of a prospective association of work-related SVH with hazardous substance use.

### SVH and sickness absence

Sickness absence, retrieved from registers, was investigated by five high quality studies from northern Europe. Four studies were conducted on national samples and one on elder care employees. Four studies investigated unwanted sexual attention or sexual harassment as a risk factor for a spell of long-term sickness absence with definitions ranging from two to eight consecutive weeks [29, 52, 53, 57]. One study investigated cumulative sickness absence of 1–16 or > 16 cumulative days [55]. These five studies found two null results, three statistically non-significant associations (one weak and two moderate) and four statistically significant associations (two weak and two strong).

In summary, results regarding work-related SVH and sickness absence are heterogeneous and there is no consistent evidence for a prospective association.

### SVH and occupational outcomes

Six included studies investigated SVH in relation to occupational outcomes. Four studies investigated turnover. One high quality study about female active US military members showed a weak association of sexual harassment with turnover [67]. One medium quality study about Danish elder care workers showed a weak to moderate association of unwanted sexual attention with turnover, though it did not follow the expected dose-response relationship [78]. One high quality study found a weak association of sexual harassment with turnover in a Swedish national sample [32]. One high quality study about women in the USA found a moderate association between severe sexual harassment and self-reported financial stress, mediated by turnover [71]. One medium quality study found no association between lifetime experiences of sexual harassment with retention in academia in female members of academic medical faculties in the USA, but severe sexual harassment was moderately predictive of advancement to full professor [63]. And one high quality study from Norway found a strong association of unwanted sexual attention with health-related employment exit in a national sample [79].

In summary, studies about occupational outcomes were too few and too diverse to draw any conclusions.

### Gender differences

Five studies were conducted on women only [61, 63, 67, 71, 78]. Seven studies included women and men but did not explore gender differences [52, 59, 65, 68, 70, 77, 79]. In total, 16 studies investigated SVH in relation to a health outcome (including sickness absence) and presented gender-stratified results or tested for multiplicative interaction of the exposure with gender in the association with the respective outcome. Of those 16 studies, four studies showed stronger associations of SVH with hypertension, substance use disorder, self-directed violence, and psychological distress in women [73–76]. Three studies showed stronger associations of SVH with sickness absence, mental distress, and use of psychiatric care in men [29, 54, 72]. The results from nine studies indicated no pronounced gender differences in the respective association [53, 57, 58, 60, 62, 64, 66, 69, 79]. None of the included studies investigating occupational outcomes explored gender differences.

In summary, there is no indication that work-related SVH affects the overall health of women and men in a substantially different magnitude.

### SVH by a member of the organization versus a third-party perpetrator

In five studies, the impact of sexual harassment from a member of the organization and a third party (e.g., customer or patient) were compared. Two studies showed similar associations of harassment from an internal and a third-party perpetrator with sickness absence and the dispense of psychotropic medication [55, 58]. Results from two studies indicated a stronger effect of harassment from a member of the organization on sickness absence and depressive symptoms [29, 66], and one study found a stronger association of harassment from a third party with suicide attempts [62].

In summary, there is no indication of a consistent difference in the health impact of sexual harassment from members of the organization or third parties.

### Behavior-based assessment versus self-labelled exposure

Overall, the ten studies that used a behavior-based approach to assess sexual harassment showed mostly null results or weak associations with the respective health outcomes [60, 64, 68–70, 77] and some strong associations [37, 59, 65], but none of moderate strength. The 11 studies that investigated self-labelled unwanted sexual attention or sexual harassment in relation to subsequent health outcomes reported mostly moderate or strong associations [29, 53–56, 58, 62] and fewer null results or weak associations [29, 52, 57, 61, 66]. However, this comparison should be interpreted with caution, as the studies also differed in country context, investigated outcomes and study designs.

## Discussion

This is the first systematic review of the occupational and health consequences of workplace GBVH, including any working population and limiting the evidence base to prospective studies, assessed as medium to high quality. We applied a broad concept of GBVH and aimed to include a broad range of potential terminology and operationalizations concerning violent or harassing behaviors towards people based on their gender or sexuality. We included 29 studies, all concerned SVH, only two studies investigated additionally non-sexual gender-based harassment. Based on these studies, we synthesized findings for SVH in relation to four health outcomes and a group of diverse occupational outcomes.

Overall, we found consistent evidence for a prospective association between work-related SVH and poor mental health. Further, there were indications of a prospective association of SVH and hazardous substance use. The results concerning SVH and subsequent sickness absence were heterogeneous and did not consistently indicate an association. Other health and occupational outcomes were insufficiently studied to draw conclusions.

Previous reviews have been mostly based on cross-sectional studies and concluded consistently that SVH is associated with self-reports of decreased occupational well-being and poor health [12, 23, 24, 30]. However, compared to other psychosocial stressors, such as e.g., job demand and control, or bullying, SVH has received relatively little attention, and the assessment of GBVH as an occupational health hazard still lacks a large body of high-quality prospective studies.

Conceptually, the research field is moving towards an integrated model of GBVH, considering SVH as one specific form of GBVH, where sexualization is mostly a means of oppression [6–10]. This was not reflected in the studies we identified. Except for two studies, the included studies focused entirely on SVH. This, even though, SVH is often experienced in combination with non-sexual displays of sexism, non-sexual gender-based harassment is far more common and may be similarly harmful [9, 12]. Resolute action against SVH is no doubt in order on all societal levels. Focusing the attention exclusively on behaviors of a sexual nature could however risk missing the struggle for heteronormative hegemony underlying a great part of SVH. In consequence, these sentiments may find their expression in more subtle behaviors, that are more difficult to point out, but may be equally harmful [18].

The victimization of men with SVH appears to have gained acknowledgement. Most studies included men, and the results indicated a similar vulnerability to SVH as in women. However, the complexity and relationality of gender was not considered in the reviewed literature. Studies conflated sex and gender and addressed gender exclusively as a binary and unambiguous identity. Studies also focused exclusively on the gender of the harassed person. Other factors that most likely are crucial for the experience, such as the gender of the perpetrator or the organizational power constellation between the harassed person and the perpetrator were seldom taken into consideration. An exception are studies from northern Europe that differentiated between harassment from members of the organization and third parties. The results from these studies indicated no consistent difference in the health impact of SVH from members of the organization and third parties on the health of subjected workers, which is an important finding considering the high prevalence and normalization of sexual harassment from third parties in some occupations. Future research should be specific in whether harassment stems from inside the organization or from third parties, as different mediating factors may be driving the associations with health, different preventive measures may be in order and different protective laws may apply.

As has been attested before, we also found a diversity in operationalizations of SVH among the included studies, and there is an obvious need for validated scales, that can be used in different contexts. The SEQ appears to have this status for the assessment of sexual harassment in the USA and was also used in some non-European countries. Still, diverse definitions and cut-offs were applied with this instrument and some authors used modified versions, without specifying the exact nature of the changes. In northern Europe, the BSHS was developed over ten years ago, but appears not to have found acceptance. Rather, the common practice was to assess self-labelled unwanted sexual attention or sexual harassment with a single item.

Self-labelling is a rather insensitive method of assessment, as it leaves the interpretation, which actions should be regarded as violence or harassment to the respondents. For sexual harassment, it is a well-established fact, that respondents only self-label a fraction of the experiences that can reasonably be considered cases [37, 80–82]. On the other hand, scales like the SEQ, with 21 items will not realistically be integrated in comprehensive longitudinal work environment surveys. Achieving continuous and standardized assessments of GBVH in longitudinal cohorts is crucial to moving the research forward, though. In fact, the selection of survey participants based on their interest in the specific subject was one of the major weaknesses of many studies that used more extensive, behavior-based exposure assessments. A short version of a validated scale in combination with a direct question about victimization as a standard instrument would be desirable. A good compromise may be a direct question in combination with a definition, containing examples, as to lower the threshold for recognizing experiences as a kind of GBVH.

Also, to strengthen the evidence about the impact of more subtle behaviors that may be considered as inconsequential, these more subtle experiences should be assessed and analyzed separately from behaviors that fall under criminal law, such as assault. And while it may be conceptually reasonable to consider GBVH as a continuum regarding severity and pervasiveness, results from continuous scales were difficult to interpret regarding the magnitude of the association with the outcome. Some studies responded to this by introducing concepts of e.g., “severe sexual harassment”. Unfortunately, this approach implied the comparison with a reference group, where a substantial part had experienced harassment. A promising approach could be the categorization into different degrees of victimization, based on meaningful thresholds regarding seriousness and pervasiveness. Furthermore, GBVH occurs within organizational power dynamics, which may be crucial for the experiences and their impact. It can e.g., be crucial if the subjected person is dealing with one perpetrator or a whole group, and if the perpetrator has authority to make relevant decisions. This can be formal authority or informal power, e.g., when someone oversees crucial resources and infrastructure. In occupations, where careers are built through strategic networking, the reputation and network of the perpetrator(s) can be highly relevant factors. It can also play an important role if co-workers are perceived as supportive, both in emotional and practical respect. As one way to escape the harassment is to exit the workplace, it may also be highly relevant if the subjected person has opportunities to transition without suffering career losses. To gain a better understanding of GBVH as an occupational health hazard, integrating these contextual factors conceptually would be highly beneficial.

With some exceptions, the temporal dimension was barely motivated in the included studies and seemed mostly data driven. In most studies, exposure status was assessed only once, and the presented results are adjusted for baseline health status. While baseline adjustment is standard procedure to account for reverse causation, it may be problematic in this context. Survey items about exposure to GBVH usually concern the past six or twelve months, and exposure before this period is entirely unknown. Unless there is reason to believe that the outcome occurs with a considerable time-lag, these results may be over-adjusted and present the health deterioration over time beyond the short-term influence of the exposure, rather than the full impact.

We found only a few longitudinal studies that addressed the temporal dimension. One study showed that the prospective association of sexual harassment with depressive symptoms was entirely explained by the elevated depression score at onset of the exposure [66], and another study showed that persistent exposure entirely explained the prospective association of prior exposure with depressive affect [60]. Two studies found prospective associations of sexual harassment with service use in individuals where the exposure had ceased and those still exposed, though the associations were stronger among those still exposed [65, 77]. It is also noteworthy that in none of the studies, where Cox proportional models were fitted, deviations were reported. This suggests that the associations were constant over the entire follow-up time (ranging from one year to 13 years). However, not all studies reported model fit analyses. Altogether, these results indicate that the impact of SVH is persistent over time, to some extent even after the exposure has ceased. More importantly, though, that no recovery was observed is most likely explained to a considerable part by the fact that exposure tends to persist. The research field would profit greatly from longitudinal studies that can further disentangle the complex relationships between people’s GBVH experiences, their occupational context, and their health status over time.

### Strengths and limitations of the review

A major strength of this review is the inclusive search strategy, which was developed in consultation with a librarian. Also, the search strategy was sent to experts from different fields and the search was conducted in databases that list records from the medical and the social sciences. Further, we ensured a systematic and transparent approach by uploading the protocol and following the PRISMA and SWiM guidelines. Through our demands for a prospective study design, minimal confounder adjustments and at least medium quality, we ensured that only evidence of reasonable reliability was included in the synthesis.

There are also some limitations to this review. First, we only included published results and only publications in English. This may have contributed to the fact that the evidence derived mainly from the USA and northern Europe, which severely limits generalizability. Also, we did not have the military as an employer in mind when we developed the search strategy and thus did not include terminology for this specific work context (e.g., “service” or “Military Sexual Trauma”). Therefore, studies concerning the military may not be sufficiently included in our review, and we cannot rule out that we overlooked further studies concerning lines of work that our search was not sensitive enough for. Considering the special character of military service as an employment, however, a review focusing exclusively on the military context would be motivated.

It could also be considered a limitation that we focused on interpersonal behavior and excluded studies that investigated formal discrimination, e.g., regarding employment, wage, or promotion. While these phenomena certainly can be related to interpersonal behaviors, this is not necessarily the case, and the mechanisms of their health impact may differ decisively. Further, we identified several potentially eligible results, but could not access enough information to include them. Moreover, the results were too diverse to conduct meta-analysis and assess reporting bias.

### Implications for future research

For the adequate assessment of GBVH as an occupational health hazard, there is a need for clear concepts of the different kinds of GBVH people experience and reliable instruments for exposure assessment. To gain the full picture, research should also comprise non-sexualizing forms of GBVH and be sensitive to the specific experiences of sexual and gender minorities. Studies should also take the organizational power constellation between the perpetrator and the target as an essential characteristic of the experience into consideration. Furthermore, the research field would profit from studies that clearly hypothesize the mechanisms of the health impact of kinds of GBVH over time and take the influence of the occupational situation into account.

## Conclusions

There are consistent evidence for work-related SVH as a risk factor for subsequent poor mental health and indications of an association with subsequent hazardous substance use. There are no indications that women and men differ regarding the health consequences of SVH, although women are more often affected than men. Research about work-related GBVH would profit from more conceptual consistency and the inclusion of non-sexual behaviors. There is a need for prospective studies that test clear hypotheses about the temporal sequence of events.

### Electronic supplementary material

Below is the link to the electronic supplementary material.

Supplementary Material 1

## Data Availability

The datasets used during and/or analysed during the current study are available from the corresponding author on reasonable request.

## Abbreviations

GBVH: Gender-based violence and harassment
ILO: International Labour Organization
SVH: Sexual violence and harassment
