# Supplementary material for: Gender-based violence and harassment at work and health and occupational outcomes. A systematic review of prospective studies
Source: BMC Public Health. 2024 Jul 4;24:1788. doi: 10.1186/s12889-024-19304-0 (PMC11225130; doi:10.1186/s12889-024-19304-0)
Supplement: Supplementary file 1 — Supplementary Material 1 [file 12889_2024_19304_MOESM1_ESM.docx]

**Additional file 1 – Appendix**

**Contents**

| Appendix 1: Deviations from the Protocol | 1 |
| --- | --- |
| Appendix 2: Full search strings | 3 |
| Appendix 3: Quality assessment tool | 4 |
| Appendix 4: Table A4. References of excluded studies and reasons for exclusion | 5 |
| Appendix 5: Table A5. Detailed description of the studies that were excluded due to low quality or repeated analyses | 10 |
| Appendix 6: Table A6. Detailed description of the studies that were included in the narrative synthesis | 12 |

**Appendix 1 – Deviations from the protocol**

The protocol is registered under Prospero CRD42023429973

***Outcomes:***

In the protocol, we stated that we include “[a]ny occupational or health outcome, self-reported or from other sources (e.g., registers), including attitudes (e.g., work satisfaction) and intentions […]”. To provide results with a clear public health relevance, we limited eligible occupational outcomes to manifest occupational outcomes and excluded measures of attitudes or intention.

***Comparators:***

We changed this criterion from “not exposed” in the protocol to “not or less exposed”, because harassment was conceptualized as a continuum in several studies and some studies applied definitions that implied higher thresholds (e.g., “severe sexual harassment”).

***Study design:***

As documented in the protocol, we originally planned to include studies with a “longitudinal or prospective design”. During screening, we realized that it is far from self-evident which designs can be regarded as longitudinal. As no consensus regarding minimal criteria for a longitudinal design, that wouldn’t already be covered by the criterium “prospective”, was reached and the literature provided no clear guidance, we changed the inclusion criterium to “prospective study design”.

We further added the inclusion criterion of a minimal adjustment for the well-established potential confounders age and gender.

***Data extraction:***

We extracted all information that was stated in the protocol and added the information if the authors reported gender differences in the association between exposure and outcome.

**Appendix 2 – Full search strings**

**Web of Science**

TS= (workplace* OR “at work” OR job* OR career* OR ”work* environment$” OR ”work* condition$” OR worker* OR employee$ OR occupation* OR "work-related” OR co-worker* OR superior* OR supervisor* OR colleague* OR third-part* OR personnel)

AND

TS= (prospective* OR cohort* OR “follow-up stud*” OR “incidence stud*” OR longitudinal* OR retrospective* OR “event stud*” OR panel$ OR subsequent OR wave$)

AND

TS= (((“unwanted sexual attention*” OR “unwanted sexual advance*”) OR (*sexual* OR ”sex-based” OR *sexis* OR *gender* OR *LGB* OR homophob* OR *gay* OR *lesbian* OR *queer* OR heteronormativ*)) NEAR/5 (abus* OR coerc* OR aggress* OR assault* OR bully* OR harm OR harass* OR hostilit* OR humiliation* OR incivilit* OR mistreatm* OR mobbing OR threat* OR violence* OR violent OR microaggression* OR discriminat* OR ”adverse behavio$r" OR ”negative act$” OR undermining OR victimization* OR policing))

AND

Pediatrics (Exclude – Web of Science Categories)

**PsycInfo**

1. (DE workplace) OR (DE ”Workplace Violence”) OR (DE ”Occupational Wellbeing”) OR (DE ”Occupational Exposure”)

2. TI (workplace* OR job* OR career* OR worker* OR employee# OR occupation* OR ”co-worker*” OR superior* OR supervisor* OR colleague* OR personnel OR (work* W1 environment#) OR (work* W1 condition*) OR (work W1 related))

3. AB (workplace* OR job* OR career* OR worker* OR employee# OR occupation* OR ”co-worker*” OR superior* OR supervisor* OR colleague* OR personnel OR (work* W1 environment#) OR (work* W1 condition#) OR (work W1 related))

4. OR/1-3

5. (DE “Sexual Harassment”) OR (DE ”Gender Violence”) OR (DE “Sex Discrimination”) OR (DE “Sexism”)

6. TI (“unwanted sexual attention” OR “unwanted sexual advance#”)

7. AB (“unwanted sexual attention” OR “unwanted sexual advance#”)

8. TI ((*sexual* OR (sex W1 based) OR *sexis* OR *gender* OR *LGB* OR homophob* OR *gay* OR *lesbian* OR *queer* OR heteronormativ*) N5 (abus* OR coerc* OR aggress* OR assault* OR bully* OR harm OR harass* OR hostilit* OR humiliation* OR incivilit* OR mistreatm* OR mobbing OR threat* OR violence* OR violent OR microaggression* OR discriminat* OR (adverse W1 behavio#r) OR ”negative act#” OR undermining OR victimization* OR policing))

9. AB ((*sexual* OR (sex W1 based) OR *sexis* OR *gender* OR *LGB* OR homophob* OR *gay* OR *lesbian* OR *queer* OR heteronormativ*) N5 (abus* OR coerc* OR aggress* OR assault* OR bully* OR harm OR harass* OR hostilit* OR humiliation* OR incivilit* OR mistreatm* OR mobbing OR threat* OR violence* OR violent OR microaggression* OR discriminat* OR (adverse W1 behavio#r) OR ”negative act#” OR undermining OR victimization* OR policing))

10. OR/5-9

11. (DE “Longitudinal studies”) OR (DE “Retrospective Studies”)

12. TI (prospective* OR cohort* OR “follow-up” OR longitudinal* OR retrospective* OR panel OR subsequent OR wave#)

13. AB (prospective* OR cohort* OR “follow-up” OR longitudinal* OR retrospective* OR panel OR subsequent OR wave#)

14. OR/11-13

15. 4 AND 10 AND 14

16. 15 filter: English, 1990 to current

17. 16 filter: excl. children <13

**Scopus**

TITLE-ABS-KEY ((*sexual* OR ”sex-based” OR *sexis* OR *gender* OR *LGB* OR homophob* OR *gay* OR *lesbian* OR *queer* OR heteronormativ*) W/5 (abus* OR coerc* OR aggress* OR assault* OR bully* OR harm OR harass* OR hostilit* OR humiliation* OR incivilit* OR mistreatm* OR mobbing OR threat* OR violence* OR violent OR microaggression* OR discriminat* OR ”adverse behavio*r” OR ”negative acts” OR undermining OR victimization* OR policing) OR ”unwanted sexual attention*” OR ”unwanted sexual advance*”)

AND

TITLE-ABS-KEY (workplace* OR ”at work” OR job* OR career* OR ”work* environment*” OR ”work* condition*” OR worker* OR employee* OR occupation* OR ”work-related” OR ”co-worker*” OR superior* OR supervisor* OR colleague* OR third-part* OR personnel)

AND

TITLE-ABS-KEY (prospective* OR cohort* OR ”follow-up stud*” OR ”incidence stud*” OR longitudinal* OR retrospective* OR ”event stud*” OR panel* OR subsequent OR wave OR waves)

NOT

EXACTKEYWORDS "Child" OR "Child Abuse" OR "Child Sexual Abuse" OR "Child Abuse, Sexual" OR "Child, Preschool" OR "Preschool Child"

**Medline**

1. exp Workplace/

2. (workplace* or at work or job* or career* or work* environment$ or work* condition$ or worker* or employee$ or occupation* or work-related or co-worker* or superior* or supervisor* or colleague* or third-part* or personnel).ti,ab,kf.

3. or/1-2

4. ((((((exp "Sexual and Gender Minorities"/) OR Perceived Discrimination/) OR Sexism/) OR Microaggression/) OR Incivility/) OR Homophobia/) OR Sexual Harassment/

5. (unwanted sexual attention* OR unwanted sexual advance*).ti,ab,kf.

6. ((sexual* or sex-based or sexis* or gender* or lgb* or homophob* or gay* or lesbian* or queer* or heteronormativ* or transsexual* or transgender or bisexual* or heterosex* or homosex*) adj5 (abus* or coerc* or aggress* or assault* or bully* or harm or harass* or hostilit* or humiliation* or incivilit* or mistreatm* or mobbing or threat* or violence* or violent or microaggression* or discriminat* or adverse behavio$r or negative acts or undermining or victimization* or policing)).ti,ab,kf.

7. or/4-6

8. exp Cohort Studies/

9. (prospective* or cohort* or follow-up stud* or incidence stud* or longitudinal* or retrospective* or event stud* or panel$ or subsequent or wave$).ti,ab,kf.

10. or/8-9

11. 3 and 7 and 10

12. exp Infant or exp Child or Pediatric*.hw

13. 11 not 12

**Appendix 3 – Quality assessment tool**


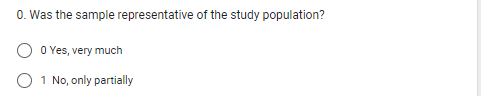


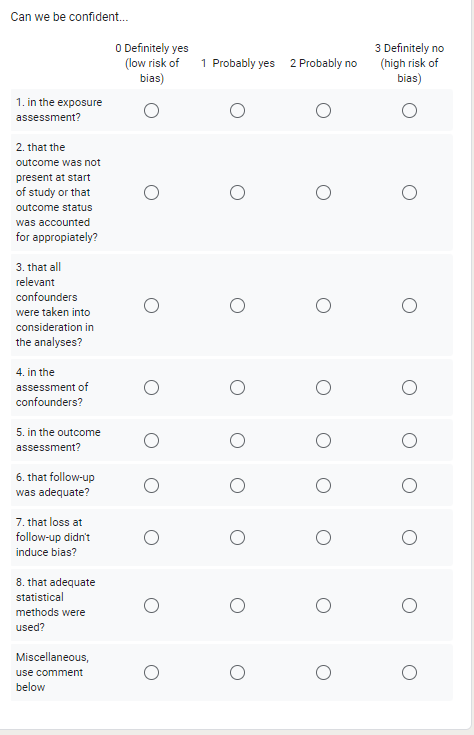


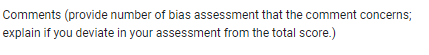


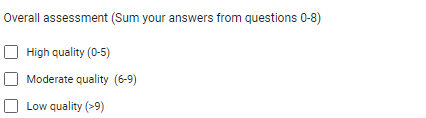


**Appendix 4 – Studies excluded with reasons**

**Reasons for exclusion**

The bold number in brackets indicates the number of studies that were excluded with this reason.

| 1. Wrong population **(1)**  2.a. Exposure not specifically gender-based or sexual **(25)**  2.b. Exposure not interpersonal or directed towards participant **(2)**  2.c. GBH is mixed with other grounds for discrimination (e.g., racism), or wage discrimination etc. **(7)**  2.d. Time of exposure unclear **(1)**  3. Work-context of exposure not clear **(12)**  4. No unexposed / less exposed comparison group **(1)** | 5. Outcome not occupational or health **(2)**  6.a. Wrong study design, not prospective **(30)**  6.b. Exposure measured retrospectively together with outcome status **(10)**  6.c. Review article **(1)**  6.d No results for direct exposure and outcome association **(5)**  7. No minimal adjustments (age & gender) **(6)**  8. Article retracted **(1)** |
| --- | --- |

**Table A4.** Studies that were screened in full-text and assessed as not eligible for inclusion in the review.

| **Nr.** | **Study** |  | **Reason** |
| --- | --- | --- | --- |
| 1 | Allen EM. Workplace discrimination and health: A longitudinal analysis of gender differences and intergenerational outcomes [dissertation]. Michigan: ProQuest Information & Learning; 2021. |  | 2.a |
| 2 | Alonso NM, O’Neill O. Going Along to Get Ahead: The Asymmetric Effects of Sexist Joviality on Status Conferral. Organ. Sci. 2022;33(5):1794-815. |  | 6.a |
| 3 | Andersen LP, Hogh A, Elklit A, Andersen JH, Biering K. Work-related threats and violence and post-traumatic symptoms in four high-risk occupations: short- and long-term symptoms. Int Arch Occup Environ Health. 2019;92(2):195-208. |  | 2.a |
| 4 | Armenta RF, Rush T, Leard Mann CA, Millegan J, Cooper A, Hoge CW, et al. Factors associated with persistent posttraumatic stress disorder among U.S. military service members and veterans. BMC Psychiatry. 2018;18(1):48. |  | 2.a |
| 5 | Azeem MU, De Clercq D, Haq IU. Suffering doubly: How victims of coworker incivility risk poor performance ratings by responding with organizational deviance, unless they leverage ingratiation skills. J Soc Psychol. 2021;161(1):86-102. |  | 2.a |
| 6 | Bell ME, Street AE, Stafford J. Victims' psychosocial well-being after reporting sexual harassment in the military. J Trauma Dissociation. 2014;15(2):133-52. |  | 4 |
| 7 | Bey GS, Person SD, Kiefe C. Gendered Race and Setting Matter: Sources of Complexity in the Relationships Between Reported Interpersonal Discrimination and Cardiovascular Health in the CARDIA Study. J Racial Ethn Health Disparities. 2020;7(4):687-97. |  | 2.c |
| 8 | Blau G, Tatum D. Correlates of perceived gender discrimination for female versus male medical technologists. Sex Roles. 2000;43(1-2):105-18. |  | 2.a |
| 9 | Blau G, Tatum DS, Ward-Cook K. Correlates of professional versus organizational withdrawal cognitions. J Vocat Behav. 2003;63(1):72-85. |  | 2.a |
| 10 | Blue B-A. Women in the work force: Job satisfaction and locus of control from 1968-1991 [dissertation] Michigan: ProQuest Information & Learning; 1996. |  | 2.a |
| 11 | Brough P, Frame R. Predicting police job satisfaction and turnover intentions: The role of social support and police organisational variables. NZ J Psychol. 2004;33(1):8-16. |  | 6.a |
| 12 | Carliner H, Sarvet AL, Gordon AR, Hasin DS. Gender discrimination, educational attainment, and illicit drug use among U.S. women. Soc Psychiatry Psychiatr Epidemiol. 2017;52(3):279-89. |  | 6.a |
| 13 | Carlson GC, Sharifian N, Jacobson IG, Leard Mann CA, Rull RP, Martin JL, et al. Contribution of post-trauma insomnia to depression and posttraumatic stress disorder in women service members: findings from the Millennium Cohort Study. Sleep. 2023;46(3). |  | 3 |
| 14 | Chang F-H. A social psychological model of women's gender-typed occupational mobility [dissertation]. Michigan: ProQuest Information & Learning; 1998. |  | 2.a |
| 15 | Chang TFH. A social psychological model of women’s gender‐typed occupational mobility. Career Dev. Int. 2003;8(1):27-39. |  | 2.a |
| 16 | Chen H, Kwan HK, Ye W-l. Effects of sexual harassment on work–family enrichment: the roles of organization-based self-esteem and Polychronicity. Asia Pac. J. Manag. 2021;40(2):409-34. |  | 5 |
| 17 | Cherry N, Arrandale V, Beach J, Galarneau JF, Mannette A, Rodgers L. Health and Work in Women and Men in the Welding and Electrical Trades: How Do They Differ? Ann Work Expo Health. 2018;62(4):393-403. |  | 6.b |
| 18 | Collinsworth LL, Fitzgerald LF, Drasgow F. In Harm's Way: Factors Related to Psychological Distress Following Sexual Harassment. Psychol Women Q. 2009;33(4):475-90. |  | 6.a |
| 19 | Farahzadi S, Rahmati M. Female labor participation in Iran: a structural model estimation. J Econ Stud. 2020;47(1):1-19. |  | 2.a |
| 20 | Farmer HS, Farmer HS. Gender differences in career development. Thousand Oaks, CA: Sage Publications, Inc; 1997. |  | 7 |
| 21 | Fillo J, Heavey SC, Homish DL, Homish GG. Deployment-Related Military Sexual Trauma Predicts Heavy Drinking and Alcohol Problems Among Male Reserve and National Guard Soldiers. Alcohol Clin Exp Res. 2018;42(1):111-9. |  | 6.a |
| 22 | Fitzgerald LF, Swan S, Fischer K. Why Didn't She Just Report Him? The Psychological and Legal Implications of Women's Responses to Sexual Harassment. J Soc Issues. 2010;51(1):117-38. |  | 6.a |
| 23 | Forel D, Vandepeer M, Duncan J, Tivey DR, Tobin SA. Leaving surgical training: some of the reasons are in surgery. ANZ J Surg. 2018;88(5):402-7. |  | 6.c |
| 24 | Foynes MM, Shipherd JC, Harrington EF. Race and gender discrimination in the Marines. Cultur Divers Ethnic Minor Psychol. 2013;19(1):111-9. |  | 2.a |
| 25 | Foynes MM, Smith BN, Shipherd JC. Associations between race-based and sex-based discrimination, health, and functioning: a longitudinal study of Marines. Med Care. 2015;53(4 Suppl 1):S128-35. |  | 6.a |
| 26 | Frank E, Carrera JS, Stratton T, Bickel J, Nora LM. Experiences of belittlement and harassment and their correlates among medical students in the United States: longitudinal survey. BMJ. 2006;333(7570):682. |  | 1 |
| 27 | Gale S, Mordukhovich I, Newlan S, McNeely E. The Impact of Workplace Harassment on Health in a Working Cohort. Front Psychol. 2019;10:1181. |  | 6.a |
| 28 | Gilmore AK, Brignone E, Painter JM, Lehavot K, Fargo J, Suo Y, et al. Military Sexual Trauma and Co-occurring Posttraumatic Stress Disorder, Depressive Disorders, and Substance Use Disorders among Returning Afghanistan and Iraq Veterans. Womens Health Issues. 2016;26(5):546-54. |  | 6.a |
| 29 | Glomb TM, Munson LJ, Hulin CL, Bergman ME, Drasgow F. Structural equation models of sexual harassment: longitudinal explorations and cross-sectional generalizations. J Appl Psychol. 1999;84(1):14-28. |  | 7 |
| 30 | Greenbaum RL, Quade MJ, Mawritz MB, Kim J, Crosby D. When the customer is unethical: the explanatory role of employee emotional exhaustion onto work-family conflict, relationship conflict with coworkers, and job neglect. J Appl Psychol. 2014;99(6):1188-203. |  | 2.a |
| 31 | Grossi PK, Bueno CH, de Abreu Silva MA, Pellizzer EP, Grossi ML. Evaluation of Sexual, Physical, and Emotional Abuse in Women Diagnosed with Temporomandibular Disorders: A Case-Control Study. Int J Prosthodont. 2018;31(6):543-51. |  | 6.a |
| 32 | Haarr RN. Factors Affecting the Decision of Police Recruits to “Drop Out” of Police Work. Police Q. 2016;8(4):431-53. |  | 6.a |
| 33 | Han X, Mortimer J, VanHeuvelen T. Perceived discrimination in the workplace and mental health from early adulthood to midlife. Longit Life Course Stud. 2022;14(1):22-47. |  | 3 |
| 34 | Hashmi SD, Shahzad K, Abbas F. The interactive effects of sexual harassment and psychological capital on victims’ burnout: evidence from the post-#MeToo movement era. Gend. Manag. 2022;37(4):509-23. |  | 7 |
| 35 | Hesson‐Mcinnis MS, Fitzgerald LF. Sexual Harassment: A Preliminary Test of an Integrative Model1. J Appl Soc Psychol. 2006;27(10):877-901. |  | 6.a |
| 36 | Hoobler JM, Rospenda KM, Lemmon G, Rosa JA. A within-subject longitudinal study of the effects of positive job experiences and generalized workplace harassment on well-being. J Occup Health Psychol. 2010;15(4):434-51. |  | 2.a |
| 37 | Hunton JE, Neidermeyer PE, Wier B. Hierarchical and gender differences in private accounting practice. Account. Horiz. 1996;10(2):14-31. |  | 8 |
| 38 | Kim S. The effect of gender discrimination in organization. Int. Rev. Public Adm. 2014;20(1):51-69. |  | 2.a |
| 39 | Kimerling R, Makin-Byrd K, Louzon S, Ignacio RV, McCarthy JF. Military Sexual Trauma and Suicide Mortality. Am J Prev Med. 2016;50(6):684-91. |  | 1 |
| 40 | Kolaja CA, Schuyler AC, Armenta RF, Orman JA, Stander VA, LeardMann CA. Sexual health difficulties among service women: the influence of posttraumatic stress disorder. J Affect Disord. 2021;292:678-86. |  | 3 |
| 41 | Latan H, Jabbour CJC, Jabbour ABLD, Ali M, Pereira V. Career satisfaction in the public sector: Implications for a more sustainable and socially responsible human resource management. Hum. Resour. Manag. J. 2022;32(4):844-63. |  | 6.a |
| 42 | Lawrence KA, Vogt D, Dugan AJ, Nigam S, Slade E, Smith BN. Mental Health and Psychosocial Functioning in Recently Separated U.S. Women Veterans: Trajectories and Bi-Directional Relationships. Int J Environ Res Public Health. 2021;18(3). |  | 6.d |
| 43 | Lesuffleur T, Chastang JF, Sandret N, Niedhammer I. Psychosocial factors at work and sickness absence: results from the French national SUMER survey. Am J Ind Med. 2014;57(6):695-708. |  | 2.c |
| 44 | de Looff P, Didden R, Embregts P, Nijman H. Burnout symptoms in forensic mental health nurses: Results from a longitudinal study. Int J Ment Health Nurs. 2019;28(1):306-17. |  | 2.a |
| 45 | Lorenz K, Kirkner A, Mazar L. Graduate Student Experiences with Sexual Harassment and Academic and Social (Dis)engagement in Higher Education. J. Women Gend. High. Educ. 2019;12(2):205-23. |  | 6.a |
| 46 | MacIntosh J, Wuest J, Ford-Gilboe M, Varcoe C. Cumulative Effects of Multiple Forms of Violence and Abuse on Women. Violence Vict. 2015;30(3):502-21. |  | 7 |
| 47 | Maguen S, Luxton DD, Skopp NA, Madden E. Gender differences in traumatic experiences and mental health in active duty soldiers redeployed from Iraq and Afghanistan. J Psychiatr Res. 2012;46(3):311-6. |  | 6.b |
| 48 | Mahon MJ, Tobin JP, Cusack DA, Kelleher C, Malone KM. Suicide among regular-duty military personnel: a retrospective case-control study of occupation-specific risk factors for workplace suicide. Am J Psychiatry. 2005;162(9):1688-96. |  | 2.a |
| 49 | Malik OF, Schat ACH, Shahzad A, Raziq MM, Faiz R. Workplace Psychological Aggression, Job Stress, and Vigor: A Test of Longitudinal Effects. J Interpers Violence. 2021;36(5-6):NP3222-NP40. |  | 2.a |
| 50 | Martinmaki SE, de Jong K, Komproe IH, Boelen PA, Kleber RJ. Incidence and Severity of Sexual Harassment, and its Impact on Mental Health in a Cohort of International Humanitarian Field-Workers. J Interpers Violence. 2023;38(11-12):7426-56. |  | 6.b |
| 51 | McCallum EB, Murdoch M, Erbes CR, Arbisi P, Polusny MA. Impact of Deployment-Related Sexual Stressors on Psychiatric Symptoms After Accounting for Predeployment Stressors: Findings From a U.S. National Guard Cohort. J Trauma Stress. 2015;28(4):307-13. |  | 6.a |
| 52 | Mensah A, Toivanen S, Diewald M, Ul Hassan M, Nyberg A. Workplace gender harassment, illegitimate tasks, and poor mental health: Hypothesized associations in a Swedish cohort. Soc Sci Med. 2022;315:115520. |  | 2.b |
| 53 | Millegan J, Milburn EK, LeardMann CA, Street AE, Williams D, Trone DW, et al. Recent Sexual Trauma and Adverse Health and Occupational Outcomes Among U.S. Service Women. J Trauma Stress. 2015;28(4):298-306. |  | 3 |
| 54 | Millegan J, Wang L, LeardMann CA, Miletich D, Street AE. Sexual Trauma and Adverse Health and Occupational Outcomes Among Men Serving in the U.S. Military. J Trauma Stress. 2016;29(2):132-40. |  | 3 |
| 55 | Moon B, McCluskey J, Morash M. Aggression against middle and high school teachers: Duration of victimization and its negative impacts. Aggress Behav. 2019;45(5):517-26. |  | 2.d |
| 56 | Munson LJ, Hulin C, Drasgow F. Longitudinal analysis of dispositional influences and sexual harassment: Effects on job and psychological outcomes. Pers. Psychol. 2000;53(1):21-46. |  | 7 |
| 57 | Murrell AJ, Olson JE, Frieze IH. Sexual Harassment and Gender Discrimination - a Longitudinal-Study of Women Managers. J Soc Issues. 1995;51(1):139-49. |  | 6.a |
| 58 | Neumark D, McLennan M. Sex Discrimination and Women's Labor Market Outcomes. J. Hum. Resour. 1995;30(4):713-40. |  | 2.a |
| 59 | Nuttbrock L, Bockting W, Rosenblum A, Hwahng S, Mason M, Macri M, et al. Gender abuse and major depression among transgender women: a prospective study of vulnerability and resilience. Am J Public Health. 2014;104(11):2191-8. |  | 3 |
| 60 | O'Hara B. Twice Penalized. J. Disabil. Policy Stud. 2016;15(1):27-34. |  | 2.a |
| 61 | Pandey N, Ashfaq SN, Dauterive EW, 3rd, MacCarthy AA, Copeland LA. Military Sexual Trauma and Obesity Among Women Veterans. J Womens Health (Larchmt). 2018;27(3):305-10. |  | 6.a |
| 62 | Panos A, Panos P, Dulle P. A 10-Year Clinical Case Study to an Incident of Workplace Violence. J. emot. abuse. 2007;4(3-4):23-47. |  | 2.a |
| 63 | Park K, Kim J. Longitudinal association between perceived discrimination and sleep problems among young adults in the United States: Tests of moderation by race/ethnicity and educational attainment. Soc Sci Med. 2023;321:115773. |  | 2.a  3 |
| 64 | Parnell D, Ram V, Cazares P, Webb-Murphy J, Roberson M, Ghaed S. Sexual Assault and Disabling PTSD in Active Duty Service Women. Mil Med. 2018;183(9-10):e481-e8. |  | 6.b |
| 65 | Pavalko EK, Mossakowski KN, Hamilton VJ. Does Perceived Discrimination Affect Health? Longitudinal Relationships between Work Discrimination and Women's Physical and Emotional Health. J. Health Soc. Behav. 2003;44(1):18-33. |  | 2.c |
| 66 | Perina DG, Marco CA, Smith-Coggins R, Kowalenko T, Johnston MM, Harvey A. Well-Being among Emergency Medicine Resident Physicians: Results from the ABEM Longitudinal Study of Emergency Medicine Residents. J Emerg Med. 2018;55(1):101-9 e2. |  | 6.a |
| 67 | Pietilainen M, Natti J, Ojala S. Perceived gender discrimination at work and subsequent long-term sickness absence among Finnish employed women. Eur J Public Health. 2020;30(2):311-6. |  | 2.b &  2.c |
| 68 | Polusny MA, Kumpula MJ, Meis LA, Erbes CR, Arbisi PA, Murdoch M, et al. Gender differences in the effects of deployment-related stressors and pre-deployment risk factors on the development of PTSD symptoms in National Guard Soldiers deployed to Iraq and Afghanistan. J Psychiatr Res. 2014;49(1):1-9. |  | 6.b |
| 69 | Reimann S, Alfermann D. Female Doctors in Conflict: How Gendering Processes in German Hospitals Influence Female Physicians' Careers. Gender Issues. 2018;35(1):52-70. |  | 6.a |
| 70 | Richman JA, Flaherty JA, Rospenda KM. Perceived workplace harassment experiences and problem drinking among physicians: Broadening the stress alienation paradigm. Addiction. 1996;91(3):391-403. |  | 6.b |
| 71 | Richman JA, Rospenda KM, Cloninger L. Terrorism, distress, and drinking: vulnerability and protective factors. J Nerv Ment Dis. 2009;197(12):909-17. |  | 6.d |
| 72 | Richman JA, Rospenda KM, Flaherty JA, Freels S. Workplace harassment, active coping, and alcohol-related outcomes. J Subst Abuse. 2001;13(3):347-66. |  | 6.b |
| 73 | Richman JA, Rospenda KM, Flaherty JA, Freels S, Zlatoper K. Perceived organizational tolerance for workplace harassment and distress and drinking over time [harassment and mental health]. Women Health. 2004;40(4):1-23. |  | 6.a |
| 74 | Richman JA, Shinsako SA, Rospenda KM, Flaherty JA, Freels S. Workplace harassment/abuse and alcohol-related outcomes: the mediating role of psychological distress. J Stud Alcohol. 2002;63(4):412-9. |  | 6.a |
| 75 | Richman JA, Wislar JS, Flaherty JA, Fendrich M, Rospenda KM. Effects on alcohol use and anxiety of the September 11, 2001, attacks and chronic work stressors: a longitudinal cohort study. Am J Public Health. 2004;94(11):2010-5. |  | 6.d |
| 76 | Rosander M, Nielsen MB. Perceived ability to defend oneself against negative treatment at work: Gender differences and different types of bullying behaviours. Appl Psychol. 2023;72(4):1430-48. |  | 2.a |
| 77 | Rosellini AJ, Street AE, Ursano RJ, Chiu WT, Heeringa SG, Monahan J, et al. Sexual Assault Victimization and Mental Health Treatment, Suicide Attempts, and Career Outcomes Among Women in the US Army. Am J Public Health. 2017;107(5):732-9. |  | 3 |
| 78 | Rospenda KM, Fujishiro K, McGinley M, Wolff JM, Richman JA. Effects of Workplace Generalized and Sexual Harassment on Abusive Drinking Among First Year Male and Female College Students: Does Prior Drinking Experience Matter? Subst Use Misuse. 2017;52(7):892-904. |  | 6.b |
| 79 | Rospenda KM, Richman JA. The factor structure of generalized workplace harassment. Violence Vict. 2004;19(2):221-38. |  | 6.a |
| 80 | Roth LM. Engendering inequality: Processes of sex-segregation on Wall Street. Sociol. Forum. 2004;19(2):203-28. |  | 6.a &  2.c |
| 81 | Seelig AD, Rivera AC, Powell TM, Williams EC, Peterson AV, Littman AJ, et al. Patterns of Smoking and Unhealthy Alcohol Use Following Sexual Trauma Among U.S. Service Members. J Trauma Stress. 2017;30(5):502-11. |  | 3 |
| 82 | Segura DA. Chicanas in White-Collar Jobs - You Have to Prove Yourself More. Sociol Perspect. 1992;35(1):163-82. |  | 6.a |
| 83 | Shinsako SA, Richman JA, Rospenda KM. Training-related harassment and drinking outcomes in medical residents versus graduate students. Subst Use Misuse. 2001;36(14):2043-63. |  | 2.c |
| 84 | Shipherd JC, Pineles SL, Gradus JL, Resick PA. Sexual harassment in the Marines, posttraumatic stress symptoms, and perceived health: evidence for sex differences. J Trauma Stress. 2009;22(1):3-10. |  | 6.a |
| 85 | Sim E, Han CS. Relumining perceived workplace gender discrimination in South Korea: examining determinants and paths through decision trees. Hum. Resour. Dev. Int. 2023. |  | 2.a &  6.a |
| 86 | Smikle CB, Fiedler E, Sorem KA, Spencer DK, Satin AJ. The impact of sexual abuse on job attrition in military recruits. Mil Med. 1996;161(3):146-8. |  | 3 |
| 87 | Smith BN, Taverna EC, Fox AB, Schnurr PP, Matteo RA, Vogt D. The Role of PTSD, Depression, and Alcohol Misuse Symptom Severity in Linking Deployment Stressor Exposure and Post-Military Work and Family Outcomes in Male and Female Veterans. Clin Psychol Sci. 2017;5(4):664-82. |  | 6.b  7 |
| 88 | Sobstad JH, Pallesen S, Bjorvatn B, Costa G, Hystad SW. Predictors of turnover intention among Norwegian nurses: A cohort study. Health Care Manage Rev. 2021;46(4):367-74. |  | 2.a |
| 89 | Stockdale MS, Logan TK, Sliter KA, Berry SA. Interpersonal Violence Victimization and Sexual Harassment: A Prospective Study of Revictimization. Sex Roles. 2014;71(1-2):55-70. |  | 6.b |
| 90 | Stockdale MS, Logan TK, Weston R. Sexual harassment and posttraumatic stress disorder: damages beyond prior abuse. Law Hum Behav. 2009;33(5):405-18. |  | 5 |
| 91 | Sumner JA, Lynch KE, Viernes B, Beckham JC, Coronado G, Dennis PA, et al. Military Sexual Trauma and Adverse Mental and Physical Health and Clinical Comorbidity in Women Veterans. Womens Health Issues. 2021;31(6):586-95. |  | 6.a |
| 92 | Templeton DM. Sexual Assault - Effects of the Research Process on All the Participants. Can. Fam. Physician. 1993;39:248-254. |  | 6.d |
| 93 | Thomas CL, Nieh C, Hooper TI, Gackstetter GD, LeardMann CA, Porter B, et al. Sexual Harassment, Sexual Assault, and Physical Activity Among U.S. Military Service Members in the Millennium Cohort Study. J Interpers Violence. 2021;36(15-16):7043-66. |  | 3 |
| 94 | Tiet QQ, Leyva YE, Blau K, Turchik JA, Rosen CS. Military sexual assault, gender, and PTSD treatment outcomes of U.S. Veterans. J Trauma Stress. 2015;28(2):92-101. |  | 6.d |
| 95 | Torok E, Hansen AM, Grynderup MB, Garde AH, Hogh A, Nabe-Nielsen K. The association between workplace bullying and depressive symptoms: the role of the perpetrator. BMC Public Health. 2016;16:993. |  | 6.a &  2.a |
| 96 | Ullman SE, Brecklin LR. Sexual assault history and health-related outcomes in a national sample of women. Psychol. Women Q. 2003;27(1):46-57. |  | 3 |
| 97 | Vargas EA, Brassel ST, Cortina LM, Settles IH, Johnson TRB, Jagsi R, editors. #MedToo: A Large-Scale Examination of the Incidence and Impact of Sexual Harassment of Physicians and Other Faculty at an Academic Medical Center. J Womens Health (Larchmt).2020;29(1):13-20. |  | 6.a |
| 98 | Wolff RJ. Microaggressions against LGBT individuals: The effects on identity formation and mental wellbeing [dissertation]. Michigan: ProQuest Information & Learning; 2021. |  | 6.a |
| 99 | Xin J, Chen SM, Kwan HK, Chiu RK, Yim FHK. Work-Family Spillover and Crossover Effects of Sexual Harassment: The Moderating Role of Work-Home Segmentation Preference. J. Bus. Ethics. 2018;147(3):619-29. |  | 7 |
| 100 | Yong Kim K, Elkins Longacre T, Werner S. The Effects of Multilevel Signals on Sex Discrimination Experiences Among Female Employees. Hum. Resour. Manag. J. 2016;56(6):995-1013. |  | 2.c |

**Appendix 5 - Studies excluded due to low quality or repeated analyses**

**Table A5.** Detailed description of the studies that were excluded due to low quality or repeated analyses

| **Reference** | **Time period** | **Age**  **(years)^a^** | **Exposure^b^** | **Exposed**  **% or**  **m(SD)^c^** | **Outcome^d^** | **Cases**  **% or**  **m (SD)^c^** | **Co-variates** | **Statistical method** | **N^e^**  **(% men)** | **Effect size**  **(CI)^e^** | **♀♂^g^** | **Q^h^** |
| --- | --- | --- | --- | --- | --- | --- | --- | --- | --- | --- | --- | --- |
| ***Excluded due to repeated analyses*** | | | | | | | | | | | | |
| Random sample of university employees in 1996 (USA) | | | | | | | | | | | | |
| Rospenda et al, 2000 | Assessment of exposure 1996 and outcome 1996 and 1997; 1-year time-lag. | ≈41 (12) | Sexual harassment in the work setting last year; 19 items from modified SEQ; Cut-off: unwanted sexual attention or gender harassment experience > once or sexual coercion ≥ once; None/Onset/Remission/ Chronicity | Remission:  13%, Chronicity:  20% | Problem drinking past 12 months; compound measure; cut-off: MAST score > 4, ≥ drinking to intoxication, or ≥ 1 heavy drinking episode. | n.r. | Age, Race, Gender, occupational group, problem drinking at baseline, work stress (psych. workload, decision latitude) at baseline and follow-up, General workplace abuse at baseline and follow-up. | Logistic regression | 1 350 (≈47) | remission:  OR 1.27 (0.83–1.95)  chronicity:  OR 1.30 (0.87–1.94) | n.t. | M  6 |
| Freels et al, 2005 | Exposure and outcome assessed 1996, 1997, 2001, and 2002. Any two adjacent waves used for exposure and subsequent outcome. | n.r. | Sexual harassment in the work setting last year; 19 items from modified SEQ; continuous standardized scale. | m 0  SD 1 | Frequency of intoxication last 12 months, one item with definition; standardized scale. | m 0  SD 1 | Age, race, years of education, outcome status at baseline, wave pair. | Mixed effects linear regression models | 999 (0) | 0.062  (SE 0.008)  P<0.05 | ♀ | M 9 |
|  |  |  |  |  |  |  |  |  | 868 (100) | 0.004  (SE 0.88) | ♂♂ |  |
|  |  |  |  |  | Alcohol dependency; MAST score; continuous standardized scale. |  |  |  | 998  (0) | 0.051  (SE 0.04) | ♀♀ |  |
|  |  |  |  |  |  |  |  |  | 870 (100) | -0.002  (SE 0.94) | ♂♂ |  |
|  |  |  |  |  | Number of drinks past month; calculated from number of drinking days and usual number of drinks; continuous standardized scale. |  |  |  | 860 (0) | -0.011  (SE 0.48) | ♀♀ |  |
|  |  |  |  |  |  |  |  |  | 819 (100) | -0.011  (SE 0.60) | ♂♂ |  |
| Richman, 2006 | Exposure assessed 1996, 1997, 2001 and only most recent observation used. Outcome assessed 2002. Time-lag 1-6 years. | n.r. | Sexual harassment in the work setting in the last year, 19 items from modified SEQ; continuous scale, mean-centered. | m 0  SD 1 | Drinking days/month; continuous scale; mean-centered. | m 7.3 (SD 8.5) | Gender, age, race, educational attainment, employment status (retiree vs. worker), baseline (1996) outcome status | Linear regression | 1 654 (44) | n.r.  p>0.05 | n.t. | M 6 |
|  |  |  |  |  | Alcoholic drinks/day; continuous scale; mean-centered. | m 1.8 (SD 1.2) |  |  | 1 654 (44) | 0.053  (SE 0.04) | n.t. |  |
| McGinley et al, 2011 | Exposure assessed repeatedly 1996-2005, outcome 2007. | n.r. | Chronic sexual harassment in the work setting over 10 years determined by latent trajectory class modelling; 19 items from modified SEQ. Categories: infrequent / chronic | 32.5% | Drinking quantity past 30 days; one item; continuous, normalized. |  | age, ethnicity, sex, outcome status 2006 | Linear regression | 2 265 (n.r.) | 0.048 p=0.004 | n.t. | M 9 |
|  |  |  |  |  | Drinking to intoxication past 12 months, continuous; normalized. |  |  |  |  | 0.032 p=0.017 | n.t. |  |
|  |  |  |  |  | Alcohol dependence past 12 months; MAST score; continuous, transformed to normalize. |  |  |  |  | 0.034  p>0.05 | n.t. |  |
|  |  |  |  |  | Binge drinking past 12 months; one item, continuous, normalized. |  |  |  |  | 0.022  p>0.05 | n.t. |  |
| ***Excluded due to low quality*** | | |  |  |  |  |  |  |  |  |  |  |
| Caregivers in well fare facilities for the elderly (JP) | | | | | | | | | | | | |
| Taniguchi, 2016 | Exposure assessed 2009, outcome 2011; 2-year time-lag. | 37 (12) | Sexual harassment in the work context past 6 months; 3 items from NAQ; 0/≥ 1 experience.  Never/onset /remission/chronic. | Remission:  15%: Chronic: 12% | High psychological stress reaction; items from BJSQ  Cut-off in men: >13, in women >12 | 19% | gender, age, job carrier, type of occupation, marital status, employment status, work shift, smoking status | Logistic regression | 543 (26) | Remission: OR 0.88 (0.42- 1.84)  Chronic:  OR 1.42  (0.42-4.79) | n.t. | L  >9 |
|  |  |  |  |  | High physical stress reaction, BJSQ  Cut-off in men: >4, in women: >5 | 18% |  |  |  | Remission: OR 0.23  (0.07-0.75), Chronic:  OR 1.16  (0.31-4.26) | n.t. | L  >9 |
| Worker Well-being survey (MX)  All supply chain workers in two apparel factories. | | | | | | | | | | | | |
| Weziak-Białowolska, 2020 | Two surveys 2017 & 2018; 1-year time-lag. | ≈ 32 (10) | Sexual harassment [harasser n.r.] last 12 months "inspired by SEQ”; [cut-off n.r.]; no/yes | ≈ 7.2-8.8.% | Intent to leave within next six months; one item; no/yes | n.r. | gender, age, marital status, education, job tenure, factory, job control, job demand, worrying about being able to meet normal monthly living expenses and safety, housing and food and prior outcome | Logistic regression | 953  (≈ 52-58) | OR 1.771  p>0.05 | n.t. | L >9 |
|  |  |  |  |  | Limited ability days [time n.r.]; one item; 0 />0. | n.r. |  |  |  | OR 2.910  p<0.001 | n.t. | L  >9 |
| *Center for Maternal and Infant Outcomes Research in Translation COMFORT (USA)*  Pregnant military service veterans who enrolled in VHA. | | | | | | | | | | | | |
| Gross & Kroll-Desrosiers et al, 2020 | Exposure assessed (baseline) 01/2016 – 09/2018 when registered as pregnant patient; follow-up 12 weeks after delivery- | ≈33 (5) | Military sexual trauma (harassment); VHA screening; one item with definition; ; no/yes. | ≈52% | Symptom severity of postnatal depression; EPDS, continuous scale. | m 5.06 (SD 5.58) | race, marital status, living status, service-connected status, prenatal stress | Linear regression | 363 (0) | b coef. 1.89 (SE 0.48)  p<0.001 | ♀♀ | L  >9 |
|  |  |  | Military sexual trauma (assault); VHA screen; one item with definition; no/yes. | ≈30% | Symptom severity of postnatal depression; EPDS, continuous scale. | m 5.06 (SD 5.58) |  |  | 358 (0) | b coef. 1.16  (SE 0.55)  p<0.01 | ♀♀ | L  >9 |

^a^ If nothing else is stated, mean age and (standard deviation). If only a percentage for age groups was reported, this is presented. Where the exact number for the analytical sample was not retrievable (not reported and not delivered on request), percentages as reported for the whole study sample are presented if available. This is indicated by the symbol ≈.

^b^ Instruments for exposure assessment: ISH=Inventory of Sexual Harassment, SEQ=Sexual harassment questionnaire, BSH=The Bergen Sexual Harassment Scale, VHA screen: Veterans Health Administration screening instrument, NAQ=Negative Acts Questionnaire.

^c^ Mean (standard deviation) for continuous measures, percent for categorical measures.

^d^ Instruments for outcome assessment: MDI = Major Depressive Inventory; CESD= Center for Epidemiologic Studies Depression Scale; BJSQ= Brief Job Stress Questionnaire; RAPI = Rutgers Alcohol Problems Index, ICD = International Statistical Classification of Diseases and Related Health Problems; EPDS = The Edinburgh Postnatal Depression Scale; GWS= General Well-being Scale of the Current Health Insurance Study Mental Health Battery; HSCL = Hopkins Symptoms Checklist

^e^ Number of individuals in the analytical sample. Where the exact number for the analytical sample was not retrievable (not reported and not delivered on request), percentages as reported for the whole study sample are presented if available. This is indicated by the symbol ≈.

^f^ If nothing else is stated, numbers in brackets are the 95% confidence interval; SE = standard error, OR= odds ratio

^g^ Difference in the association between the exposure and outcome among women versus among men; n.t. = not tested; ♀=♂ = no gender difference found; ♀+ = stronger association in women ♂+ = stronger association in men; ♀♀ = only women in the sample; ♂♂ = only men in the sample.

^h^ Quality assessment and total score; M = Medium quality (6-9); H = High quality (0-5); L=Low (>9).

**Appendix 6 – Detailed description of included studies**

**Table A6.** Detailed description of the studies that were included in the narrative synthesis, sorted by cohort and within cohort by date of publication.

|  | **Reference** | **Time period** | **Age**  **(SD)^a^** | **Exposure^b^** | **Exposed**  **% or**  **m(SD)^c^** | **Outcome^d^** | **Cases**  **% or**  **mean (SD)^c^** | **Exclusion criteria**  **and co-variates** | **Statistical method** | **n^e^**  **(% men)** | **Effect size**  **(CI or SE)^f^** | **♀♂^g^** | **Q^h^** |
| --- | --- | --- | --- | --- | --- | --- | --- | --- | --- | --- | --- | --- | --- |
| Random sample of university employees in 1996 (USA) | | | | | | | | | | | | | |
| 1 | Wislar et al, 2002 | Assessment of exposure 1996 and outcome 1996 and 1997; 1-year time-lag. | ≈41 (12) | Sexual harassment in the work setting last year; 19 items from modified SEQ,  Cut-off: unwanted sexual attention or gender harassment experience > once or sexual coercion ≥ once; None/Onset/Remission/ Chronicity | Rem.:  14%  Chron.  20% | Problem drinking past 12 months; MAST score; <4/≥4 | n.r. | Individuals without lifetime alcohol consumption excluded. Adjusted for age, gender, outcome status at baseline, Race/Ethnicity, Occupation, narcissism, ego strength. | Logistic regression | 1 433 (≈47) | Remission:  OR 1.46  (0.94–2.26)  Chronicity  OR 1.35  (0.91–2.01) | n.t. | M 6 |
| 2 | Rospenda et al, 2006 | Exposure assessed 1996, 1997 and 2001, outcome 2001. 3-year time-lag | n.r. | Sexual harassment in the work setting last year; 19 items from modified SEQ,  Cut-off: unwanted sexual attention or gender harassment experience > once or sexual coercion ≥ once; None/Onset/Remission/ Intermittent/Chronic | Rem.:  ≈22%,  Chron.:  ≈18 %  Inter.:  ≈4% | Services use past 3 years to deal with work-stress; one item; battery of health care or non-health professionals as response options. | 26% | Adjusted for gender, race, occupational group, age, marital status, psychological demand and decision latitude, and services use 1996. | Logistic regression | ≈1 368 (44) | Remission:  OR 1.57  (1.06–2.32)  Intermittent:  OR 2.87  (1.24–6.65), Chronic:  OR 2.56  (1.75–3.75) | ♀=♂ | M 8 |
| *U.S. Department of Defense survey of gender relations in the armed forces (USA)*  Active members of US military service | | | | | | | | | | | | | |
| 3 | Sims et al, 2005 | Exposure assessed by survey 1995, outcome continuous follow-up in registers until 09/1999. | n.r. | Sexual harassment by supervisors or coworkers past year; SEQ–DoD, 23 items; continuous scale | Mean 0.38 (0.51)  ≥1 event:  76.5% | Turnover (leaving the military); administrative records. | 13% | paygrade rank, marital status, organization commitment | Cox proportional hazards models | 11 521 (0) | HR: 1.09  (n.r.)  p<0.01 | ♀♀ | H 5 |
| National employed adults’ harassment experiences cohort (USA)  Random sample of ≥18-year-old, employed residents | | | | | | | | | | | | | |
| 4 | Shannon et al, 2007 | Exposure assessed 2003/4, outcome 2004/5; 1-year time-lag. | ≈43 (12) | Sexual harassment at the job past 12 months; modified SEQ, 9 items; cut-off: unwanted sexual attention or gender harassment experience > once or sexual coercion ≥ once.  None / Chronic / Remission / Onset | Chronic: 19%  Remission: 15% | Service use past 12 months to deal with work-stress ; one item; battery of health care or non-health professionals as response options. | ≈18% | age, income, gender, race, education, life stressors, change in life stressors, job stressors, change in job stressors, baseline services use. | Logistic regression | 1 196 (≈53) | Chronic:  OR 1.45  (0.94–2.23)  Remission:  OR 1.16  (0.72–1.89) | n.t. | M 6 |
| 5 | Rospenda et al, 2008 | As above | n.r. | Sexual harassment at the job past 12 months; modified SEQ, 9 items; cut-off: unwanted sexual attention or gender harassment experience > once or sexual coercion ≥ once. | 51.8% | Frequency of heavy episodic drinking (5+drinks on the same occasion) (time not reported); one item; count. | 20% >0 | age, race/ethnicity, occupation, education, household income, census region, number of phone lines in the household, number of eligible individuals in the household, perceived job stress, stressful life events, outcome status at baseline. | Negative binomial regression | 722 (0) | IRR 1.13  (0.93-1.37) | ♀♀ | M 7 |
|  | As above | As above | n.r. | Sexual harassment at the job past 12 months; modified SEQ, 9 items; cut-off: unwanted sexual attention or gender harassment experience > once or sexual coercion ≥ once. | 39.7% | Frequency of heavy episodic drinking; one item; count. | 38% >0 | As above | As above | 733 (100) | IRR 1.41  (1.10-1.57) | ♂♂ | M 7 |
| *Youth Development Study (YDS) (USA)*  Public school 9^th^-graders (age 14–15) in 1988 | | | | | | | | | | | | | |
| 6 | Houle et al, 2011 | Baseline 1988, exposure assessed 2003, outcome 2004; 1-year time-lag. | 30-31 | Sexual harassment by a supervisor, co-worker, customer, or client past year. 7 items from ISH and SEQ; harassment scale considering severity and frequency created with item-response theory (IRT) scaling. | mean 0.04 (1.85) | Depressive affect; 4 items from GWS; continuous scale.  Mean: 9.2 (13.1) | mean 9.2  (13.1) | Gender, race, at age 30/31: education, marital/cohabiting status, employment status, at age 14/15: household income, depressive affect | OLS (linear regression) | 732  (42) | b coef 0.22  (SE 0.06)  p<0.001 | ♀=♂ | H 5 |
| 7 | McLaughlin et al, 2017 | Exposure assessed 2003, outcome 2005. | 29-30 | Severe sexual harassment supervisor, co-worker, customer, or client past year, meeting legal definitions of hostile work environment; 7 items from ISH and SEQ.  Cut-off: At least unwanted touching and/or experiencing ≥4 different harassing behaviors. no/yes | 10.7% | financial stress past year; one item; 7-point scale; continuous. | mean 4.34 (1.78) | supervisory authority, work hours, logged employees, industry percent women, temporary job, long-term career job, race, years education, marital/cohabiting status, motherhood, a recent birth (2003-2005), negative life events (2003-2005) | linear regression | 364 (0) | b coef. 0.72  (SE 0.32)  p ≤ 0.05 | ♀♀ | H 4 |
| Employees in elder-care services (DK) | | | | | | | | | | | | | |
| 8 | Clausen et al, 2012 | Exposure in survey 2004/05, 1-year continuous follow-up in registers. | 45 (10) | Unwanted sexual attention at work past 12 months; one item, no definition; never/occasional/frequent. | Occas.:  8.3%  Frequ.:  1% | Sickness absence ≥ 8 consecutive weeks; register-based | 6.2% | Men excluded. Adjusted for age, job function, tenure, BMI, smoking status, psychosocial work conditions | Cox proportional hazards models | 9520 (0) | Occasional:  HR 0.99  (0.74–1.32)  Frequent:  HR 1.46  (0.75–2.82) | ♀♀ | H 5 |
| 9 | Clausen et al, 2013 | Exposure assessed 2004/05, outcome 2006; ≈ 1-1 ½-year time-lag. | 43 (8) | Unwanted sexual attention at work past 12 months; one item, no definition; never/occasional/frequent. | Occas.:  ≈10%, Frequ.:  ≈2% | Turnover, register-based and survey item. | 12% | Individuals age > 55 years or not directly engaged in care provision excluded. Adjusted for age, gender | Logistic regression | 4826 (≈2) | Occasional:  OR 1.33  (1.03–1.71)  Frequent:  OR 1.06  (0.58–1.93) | n.t. | M 7 |
| The Bergen Sexual Harassment Scale cohort (NO)  Random sample of 18-67 year-olds, employed at organizations with ≥5 staff, mean working time >15hours/week in ≥ 6 months before baseline. (NO) | | | | | | | | | | | | | |
| 10 | Nielsen et al, 2012 | Assessment of exposure 2005 and outcome 2007; 2-year time-lag. | 45 (n.r.) | Sexual harassment at present workplace or work-related social event last 6 month; 11 items from BSHS; no / ≥1 experience | 17% | Psychological distress; HSCL-25; <1.75/≥1.75 | 14% | Age, psychological distress at baseline. | Logistic regression | 976 (0) | OR 2.03  (1.2–3.39) | ♀♀ | H 4 |
|  | As above | As above | 46 (n.r.) | As above | 18% | As above | 12% | As above | As above | 799 (100) | OR 1.32  (0.72–2.43) | ♂♂ | H 4 |
| Individuals who took part in recruit training for the Marines in 1997 (USA) | | | | | | | | | | | | | |
| 11 | Gradus et al, 2013 | 1997 surveys at start and after 13-week recruit training, follow-up survey 10 years later and registers. | 19 (2) | Sexual harassment during recruit training; SEQ; 0/≥ 1 experience. | 19% | Attempted Suicide; one-item and register. | 3.6% | Unit cohesion, recruit training stress, graduation from recruit training, depressive symptoms, PTSD, anxiety symptoms, suicide attempt during recruit training, mental health at start of recruit training (gender tested and omitted). | Logistic regression | 646 (≈46) | OR 2.8  (1.2-6.6) | n.t. | M  8 |
| *Danish Work Environment Cohort Study, (DWECS-2000 and DWECS-2005) & Copenhagen Psychosocial Questionnaire Study (COPSOQ-II)* (DK)  Three random samples from residents at working age. | | | | | | | | | | | | | |
| 12 | Hogh et al, 2016 | Surveys conducted 2000, 2004 and 2005, used as baseline, 18 months continuous follow-up in registers. | 41 (11) | Unwanted sexual attention from colleagues, managers and/or subordinates past 12 months, one item, no definition; no/yes. | 1.3% | Incidence long-term sickness absence; < 15 / ≥ 15 consecutive days, register-based. | 9.9% | Individuals with outcome at baseline excluded.  Adjusted for age, mode of interviewing, occupational group, influence at work, work pace | Cox proportional hazards models | 9 599 (0) | HR 1.10  (0.60-2.00) | ♀♀ | H 5 |
|  | As above | As above | 42 (11) | As above | 0.7% | Definition as above | 8.4% | As above | As above | 9 767 (100) | HR 2.47  (1.32-4.65) | ♂♂ | H 5 |
|  | As above | As above | 41 | Unwanted sexual attention from clients/customers/patients in the past 12 months, one item, no definition. | 1.7% | Definition as above. | 9.9% | As above | As above | 9 599 (0) | HR 0.89  (0.52-1.51) | ♀♀ | H 5 |
|  | A above | A above | 42 | As above | 1.0% | Definition as above. | 8.4% | As above | As above | 9 767 (100) | HR 1.31  (0.67-2.54) | ♂♂ | H 5 |
| *Workplace Bullying and Harassment (WBH) cohort & Psychosocial Risk Factors for Stress and Mental Disease (PRISME) cohort*  Employees in any occupation (WBH), hospital and civil-service employees (PRISME) (DK) | | | | | | | | | | | | | |
| 13 | Nabe-Nielsen et al, 2016 | Three survey waves (2006, 2008 and 2011) as baseline, two-year continuous follow-up in registers. | 48 (10) | Unwanted sexual attention at work past 6 (PRISME) or 12 (WBH) months; one item with a definition; no/yes, ≥ one time. | 2.7% | Long-term sickness absence, register-based.  <30/≥30 consecutive days. | ≈ 9-12% | Individuals with outcome in previous 2 years excluded. Adjusted for survey, age, gender, education, alcohol consumption, smoking, BMI | Logistic regression | 8 669 observa-tions (≈30) | OR 1.61  (1.11–2. 41) | ♀=♂ | H 4 |
| Random samples of first-year students at 8 colleges and universities (USA) | | | | | | | | | | | | | |
| 14 | Wolff et al, 2017 | Exposure assessed 2011, outcome 2012, ≈6- months time-lag. | ≈19 (2) | Sexual harassment from bosses, co-workers, or customers/clients past 12 months; 13 items from SEQ; continuous. | Mean n.r. (n.r.) | Depressive symptoms past week, seven items from CESD, count. | Mean n.r. (n.r.) | Not adjusted for covariates. Participants were similar in age and analyses stratified for gender. | two-wave cross-lagged panel models | 925 (0) | 0.03  (SE 0.01)  p<0.05 | ♀♀ | M 5 |
|  | As above | As above |  | As above | Mean n.r. (n.r.) | As above | Mean n.r. (n.r.) | As above | As above | 640 (100) | 0.04 (SE 0.03)  p>0.05 | ♂♂ | M 5 |
|  | As above | As above | As above | As above | Mean n.r. (n.r.) | Alcohol-related problems, RAPI score, continuous. | Mean n.r. (n.r.) | As above | As above | 926 (0) | b coef logit:  0.14 (SE 0.10)  p>0.05  b coef count:  0.07 (SE 0.02)  p<0.01 | ♀♀ | M 5 |
|  | As above | As above | As above | As above | Mean n.r. (n.r.) | As above | Mean n.r. (n.r.) | As above | As above | 640 (100) | b coef logit:  -0.13 (SE 0.25)  p>0.05  b coef. count:  0.09 (SE 0.04)  p<0.05 | ♂♂ | M 5 |
| Veterans of military service in Iraq and Afghanistan who enrolled with VHA (USA) | | | | | | | | | | | | | |
| 15 | Brignone et al, 2017 | Assessment of exposure at first encounter with VHA clinic, 5-year continuous follow-up in registers. | ≈32 (9) | Military sexual trauma (harassment or assault); VHA screen; two items with definition; no/yes. | 2.6% | Use of outpatient mental health care over 5-year period; register-based. | 9.4 % | Gender, age, education, marital status, race/ethnicity), rank, component (Active Duty, Reserve, Guard), branch of service. | Negative binomial regression | 485 884 (88) | OR 2.82  (2.62-3.05) | ♂+ | M 9 |
|  | As above | As above | As above | As above | As above | Use of inpatient psychiatric care over 5-year period. Register-based. | 0.6% | As above | Negative binomial regression | 485 884 (88) | OR 2.57  (2.30-2.87) | ♂+ |  |
|  | As above | As above | As above | As above | As above | Use of inpatient substance use care over 5-year period; register-based | 0% | As above | Negative binomial regression | 485 884 (88) | OR 1.73  (1.22-2.44) | ♀=♂ |  |
|  | As above | As above | As above | As above | As above | Use of outpatient substance use care over 5-year period; register-based | 0.2 | As above | Negative binomial regression | 485 884 (88) | OR 2.12  (1.91-2.36) | ♀=♂ |  |
| 16 | Goldberg et al, 2019 | Assessment of exposure at first VA clinic encounter, (2001-2009), continuous follow-up for ≥ 5 years. | ≈32(9) | Military sexual trauma (harassment or assault); VHA screen; two items with definition; no/yes. | 3.7% | Alcohol use disorder; register-based (ICD codes) | 12% | race, branch of service, combat exposure, age, gender, interaction between MST and gender | Logistic regression | 390 833 (≈87) | OR 1.63  (1.49-1.79) | ♀+ | M 9 |
|  | As above | As above | As above | Military sexual trauma (harassment or assault); VHA screen; two items with definition; no/yes. | 3.8% | Drug use disorder; register-based (ICD codes) | 10% | As above | As above | 435 690 (≈87) | OR 2.26 (2.09-2.43) | ♀+ |  |
| 17 | Gaffey et al, 2022 | Assessment of exposure at first VA clinic encounter (2001-2017), continuous follow-up (mean: 10 years). | 32 (9) | Military sexual trauma (harassment or assault); VHA screen; two items with definitions; no/yes. | 5% | Incident hypertension; diagnosis or treatment for hypertension; register-based. | 9% | Individuals with outcome at baseline excluded. Adjusted for age, sex, race, ethnicity, lifestyle factors (obesity, smoking, drug use, alcohol abuse), cardiovascular factors (SBP and DBP at baseline, lipid disorders, diabetes), psychiatric diagnoses (major depressive disorder, general anxiety disorder, PTSD). | Cox proportional hazard models | 788 161 (87) | HR 1.15  1.11–1.19) | ♀+ | M 6 |
|  | As above | As above | 32 (8) | As above | 23.4% | As above | 11% | As above | As above | 100 370 (0) | HR 1.20  (1.15–1.26) | ♀♀ | M 6 |
|  | As above | As above | 32 (9) | As above | 1.7% | As above | 9% | As above | As above | 687 791 (100) | HR 1.06  (1.00–1.12) | ♂♂ | M 6 |
| Frontline service workers in hotels (CN) | | | | | | | | | | | | | |
| 18 | Zhu et al, 2018 | Exposure and outcome assessed in two surveys with 1-month time-lag. Year n.r. | 20-30:  64%  30-50:  34%  >50: 2% | Sexual harassment by supervisors/coworkers/customers (time not specified); version of SEQ, 21 items; continuous scale. | 14%  ≥1 exp-erience  mean 1.32 (0.61) | Depression past week; CESD, 20 items; continuous scale. | Mean 1.99 (0.71) | Age, gender, education, tenure, company. | Linear regression | 266 (20) | b coef. 0.21 (p<0.01) | n.t. | M  8 |
| *Work Environment and Health in Denmark (WEHD) study* (DK)  Random sample of employed residents 16-84 years | | | | | | | | | | | | | |
| 19 | Rugulies et al, 2020 | Three survey waves; baseline 2012, exposure assessed 2014, outcome 2016. | 47 (9) | Sexual harassment last 12 months; one item, no definition; No exposure/exposure by non-workplace personnel (non-WP)/exposure by workplace personnel (WP). | WP:  0.5%  Non-WP:  1% | Depressive symptoms last 2 weeks; MDI, 12 items. | mean ≈7.6  (7) | Individuals reporting sexual harassment at baseline excluded. Adjusted for age, sex, cohabitation, education, occupational status, occupational sector, depressive symptoms, treatment for depression at baseline. | Linear regression | 6 647 (46.7) | non-WP:  b coef 0.76  (-0.65–2.18)  WP:  b cof. 2.54 (0.62–4.46) | ♀=♂ | M 4 |
| Representative sample of members of 24 academic medical faculties (USA) | | | | | | | | | | | | | |
| 20 | Raj et al, 2020 | Baseline survey 1995, follow-up through survey 2012/13 and public databases. | ≈45 (9) | Sexual harassment by a superior or colleague over life-time, one item with definition; 0/>0 experiences. | ≈54% | Full professor; assessed by survey item and public databases. | ≈60% | Region, department, type of institution, years since first faculty appointment, percent of time in administration, percent of time in research | Logistic regression | 494 (0) | OR 1.25  (0.80-1.95) | ♀♀ | M 8 |
|  | As above | As above | As above | As above | As above | Retention in academics. | ≈81% | Race, marital status, region, department, years since first faculty appointment | As above | 561 (0) | OR 0.69  (0.43-1.10) | ♀♀ |  |
|  | As above | As above | As above | Severe sexual harassment by a superior or colleague over life-time, several items for: unwanted sexual advances, subtle bribery or threats to engage in sexual behavior, or coercive advances; 0/≥1 experiences; No severe sexual harassment/severe sexual harassment. | ≈32% | Full professor; survey item and public databases. | ≈60% | Region, department, type of institution, years since first faculty appointment, percent of time in administration, percent of time in research. | As above | 494 (0) | OR 1.77  (1.10-2.87) | ♀♀ |  |
|  | As above | As above | As above | As above | As above | Retention in academics; survey item and public databases. | ≈81% | race (white, non-white), marital status region, department, years since first faculty appointment. | As above | 561 (0) | OR 0.93  (0.56-1.54) | ♀♀ |  |
| *Women Veterans Cohort Study (USA)*  Veterans of military service who enrolled with VHA 01/2001-09/2014. | | | | | | | | | | | | | |
| 21 | Gross, Ronzitti et al, 2020 | Assessment of exposure at first VA clinic encounter, continuous follow-up (time n.r.) | 33(10) | Military sexual trauma (harassment or assault); VHA screen; two items with definitions; no/yes.  Exposed: 4%  Male without MST/Male with MST/Female without MST/Female with MST | 4% | Nonfatal Severe Self-Directed Violence (SDV) resulting in inpatient hospitalization; ICD codes; register-based. | 1.2% | Age, education, ethnicity/race, marital status, psychiatric comorbidities. | Cox proportional hazard models | 750 176 (88) | Male with MST:  HR 1.28  (1.10-1.48)  Female without MST:  HR 1.05  (0.94-1.18)  Female with MST:  HR 1.63  (1.46-1.83) | ♀+ | M 9 |
| *Swedish Work Environment Survey (SWES)* (SE)  Random sample of residents 16–64 years old, gainfully employed and not off work three months before survey participation. | | | | | | | | | | | | | |
| 22 | Magnusson Hanson et al, 2020 | Biannual cross-sectional surveys 1995-2013 as respective baseline, continuous follow-up in registers till 2016 (mean: 13 years). | 43 (12) | Sexual harassment by a superior, colleague or third party (e.g., patients, clients, passengers, students) past 12 months; one item with definition; no/yes. | 4.8% | Suicide, register-based. | 0.1% | Individuals with previous suicide attempts excluded. Adjusted for sex, birth country, family situation, education, income, demands, control, social support at work, workplace bullying, poor mental health at baseline, age as time scale. | Cox proportional hazards models | 82 860 (≈48) | HR 2.47  (1.25- 4.87) | ♀=♂ | H 4 |
|  | As above | As above | As above | As above | As above | Suicide attempt, register-based. | 1% | As above | As above | 82 233  (≈48) | HR 1.56  (1.18- 2.05) | ♀=♂ | H 4 |
|  | As above | As above | As above | Sexual harassment by a superior or colleague past 12 months; one item with definition; no/yes. | 1.5% | Suicide, register-based. | 0.1% | As above | As above | 82 851  (≈48) | HR 1.22  (0.29 -5.09) | n.t. | H 4 |
|  | As above | As above | As above | As above | As above | Suicide attempt, register-based. | 1% | As above | As above | 82 224  (≈48) | HR 1.54  (0.97-2.46) | n.t. | H 4 |
|  | As above | As above | As above | Sexual harassment by a third party (e.g., patients, clients, passengers, students) past 12 months; one item with definition; no/yes. | 3.8% | Suicide, register-based. | 0.1% | As above | As above | 82 855  (≈48) | HR 2.93  (1.44-5.95) | n.t. | H 4 |
|  | As above | As above | As above | As above | As above | Suicide attempt, register-based. | 1% | As above | As above | 82 228  (≈48%) | HR 1.71  (1.27-2.29) | n.t. | H 4 |
| 23 | Blindow et al, 2021 | Biannual cross-sectional surveys 1999 to 2013 as respective baseline, 1 year follow-up in registers. | 16-35:  27.5%  36-55:  53.4%  56-64:  19.1% | Sexual harassment by a superior or colleague past 12 months, one item with definition. No/ once/ Monthly to daily (mo-daily). | Once:  1,5%  Mo-daily:  0.7% | Long-term sickness absence; <21 / ≥ 21 consecutive days, register-based. | ≈13.76% | Individuals with outcome  year before baseline excluded.  Adjusted for survey wave, age, parental migration background,  Adjusted for country of birth, education, family situation, income, industry classification. | Poisson regression | 28 998 (0) | Once:  RR 0.99  (0.97–1.02) Mo-daily:  RR 1.06  (1.01–1.10) | ♀♀ | H 5 |
|  | As above | As above | As above | Gender harassment by a superior or colleague past 12 months, one item with definition. Not in 12 months / Once in 12 months / Monthly to daily (mo-daily). | Once:  7.3%  Mo-daily:  4.0% | As above | ≈ 13.76% | As above | As above | 28 702 (0) | Once:  RR 1.02  (1.00–1.03)  Mo-daily:  RR 1.04  (1.02–1.05) | ♀♀ | H 5 |
|  | As above | As above | 16-35:  28.4%  36-55:  51.9%  56-64:  19.8% | Sexual harassment by a superior or colleague past 12 months, one item with definition. Not in 12 months / Once in 12 months / Monthly to daily (mo-daily). | Once:  0.5%  Mo-daily:  0.4% | As above | ≈7.45% | As above | As above | 27 588 (100) | Once:  RR 1.01  (0.97–1.05)  Mo-daily:  RR 1.05  (0.99–1.10) | ♂♂ | H 5 |
|  | As above | As above | As above | Gender harassment by a superior or colleague past 12 months, one item with definition. Not in 12 months / Once in 12 months / Monthly to daily (mo-daily). | Once:  2.5%  Mo-daily:  1.3% | As above | ≈7.45% | As above | As above | 27 417 (100) | Once:  RR 1.02  (1.00–1.04) Mo-daily:  RR 1.05  (1.02–1.08) | ♂♂ | H 5 |
| 24 | Blindow et al, 2022 | Biannual cross-sectional surveys 2007 to 2013 as respective baseline, continuous follow-up in registers till 2015 (mean: 6.4 years). | 16-35:  ≈26%  36-55:  ≈53%  56-64:  ≈21% | Sexual harassment by a superior, colleague, or third party past 12 months; one item with definition. Not in 12 months / Once in 12 months / Monthly to daily (mo-daily). | Once:  3.6%  Mo-daily:  1.7% | Incidence dispense of psychotropic medication (antidepressants, anxiolytics, hypnotics/sedatives); register- based. | 18.5% | Individuals with outcome during baseline year excluded. Adjusted for survey year, gender, age, education, family situation, income, parental  migration background, managerial responsibilities, industry classification. | Cox proportional hazards models | 22 467 (≈49) | Once:  HR 1.15  (0.99 to 1.33)  Mo-daily:  HR 1.37  (1.12 to 1.67) | ♀=♂ | H 4 |
|  | As above | As above | As above | Sexual harassment by a superior or colleague past 12 months; one item with definition. no/once /monthly to daily (mo-daily). | Once:  0.9%  Mo-daily:  0.6% | As above | 18.5% | As above | Cox proportional hazards models | 22 467 (≈49) | Once:  HR 1.13  (0.85-1.51)  Mo-daily:  HR 1.40  (1.00-1.96) | ♀=♂ | H 4 |
|  | As above | As above | As above | Sexual harassment by a third party (e.g., patients, clients, passengers, students) past 12 months; one item with definition. Not in 12 months / Once in 12 months / Monthly to daily (mo-daily). | Once:  2.9%  Mo-daily:  1.3% | As above | As above | As above | As above | 22 467 (≈49) | Once:  HR 1.14  (0.98-1.34)  Mo-daily:  HR 1.39  (1.12-1.73) | ♀=♂ | H 4 |
|  | As above | As above | As above | Gender harassment by a superior or colleague past 12 months; one item with definition. Not in 12 months / Once in 12 months / Monthly to daily (mo-daily). | Once:  5.4%  Mo-daily:  2.5% | As above | As above | As above | As above | 22 467 (≈49) | Once:  HR 1.23  (1.09 -1.39)  Mo-daily:  HR 1.17  (0.98-1.40) | ♀=♂ | H 4 |
|  | As above | As above | n.r. | As above | Once:  7.7%  Mo-daily:  3.8% | As above | 22.8% | As above | As above | 11 436 (0) | Once:  HR 1.19  (1.04-1.37)  Mo-daily:  HR 1.14  (0.93-1.38) | ♀♀ | H 4 |
|  | As above | As above | n.r. | As above | Once:  2.9%  Mo-daily:  1.2% | As above | 14.0% | As above | As above | 11 031  (100) | Once:  HR 1.31  (1.01-1.70)  Mo-daily:  HR 1.23  (0.81-1.88) | ♂♂ | H 4 |
| 25 | Folke et al, 2022 | Biannual cross-sectional surveys 1999 to 2007 as respective baseline, 3-years prior and 3-year follow-up in registers. | 41.7.1 | Sexual harassment by a superior or colleague past 12 months; two items with definitions of sexist and sexual hostility and unwanted sexual attention, no/yes. | 12.5% | Turnover within three years, register-based. | 18.6% | Individuals working in workplaces with under 5 employees ,>60 years old, or unemployed during follow-up excluded, adjusted for survey year, age, education, region of birth, marital and parental status, workplace share of men | difference-in-differences (ordinary least squares) | 17 971 | ATET 4.15%  P= 0.000 | ♀♀ | H 4 |
|  | As above | As above | 41.5 | As above | 4.1% | As above | 20.8% | As above | As above | 15 486 | ATET 3.54%  P=0.052 | ♂♂ | H 4 |
| *The Survey of Level of Living-Working Conditions* (NO)  Random sample of residents, 18-66 years old, gainfully employed at baseline. | | | | | | | | | | | | | |
| 26 | Sterud, Degerud et al, 2021 | Exposure assessed in three surveys (2009- 2017) as baseline, 1-year continuous follow-up in registers. | 17-34: ≈28%  35-49: ≈41%  50-66:  ≈31% | Unwanted sexual attention at the workplace; one item; no/yes, ≥1 experience a month. | 3.7% | Cumulative sick leave days/calendar year; register-based; 0/1–16 (low level, (LLSL))/>16 (high level (LSL). | LLSL: 18.4%  HLSL: 16.1% | sex, age, number of actual working days, occupation, education level, sick leave days in baseline year. | mixed effects logistic regression | LLSL:  18 179 HLSL: 17 685  Observations (≈52) | LLSL:  OR 1.35  (1.09 to 1.67)  HLSL:  OR 1.41  (1.10 to 1.79) | ♀=♂ | H 4 |
|  | As above | Exposure assessed 2016, 1-year continuous follow-up in registers. | n.r. | Unwanted sexual attention by co-worker/leader; one item; no/≥1 experience a month. | n.r. | Cumulative sick leave days/calendar year; register-based; 0/>0. | n.r. | n.r. | n.r. | n.r. | OR 1.40  (0.88 to 2.22) | n.t. |  |
|  | As above | As above | n.r. | Unwanted sexual attention by client/customer; one item; no/yes, ≥1 experience a month. | n.r. | As above | n.r. | n.r. | n.r. | n.r. | OR 1.71  (1.31 to 2.24) | n.t. |  |
| 27 | Sterud, Hanvold et al, 2021 | Two survey waves,  2013/14 and 2016/17; ≈ 3-year time-lag. | 18-34:  26%  35-49:  41%  50-66:  33% | Unwanted sexual attention at the workplace; one item; no/yes, ≥1 experience a month. | 4.9% | Mental distress last 2 weeks; HSCL-5; <2.0/≥2.0. | 6.6% | sex, age, occupation, education level, mental distress at baseline | Logistic regression | 3654 (51) | OR 1.64 (1.03−2.61) | ♂+ | H 4 |
|  | As above | As above | n.r. | As above | 8.0% | As above | 8.6 | n.r. | As above | 1798 (0) | OR 1.37 (0.81−2.32) | ♀♀ |  |
|  | As above | As above | n.r. | As above | 1.8% | As above | 4.7 | n.r. | As above | 1856 (100) | OR 3.83  (1.54-9.51) | ♂♂ |  |
| 28 | Sterud et al, 2023 | Four survey waves (2006-2016); observations from two consecutive surveys baseline and follow-up. | 17-34:  26%  35-49:  41%  50-66:  33% | Unwanted sexual attention at the workplace; one item; no/yes, ≥1 experience a month. | 3.7% | Health-related employment exit; one item. | 2.6% | sex, age, weekly working hours, education level, occupation | Mixed-effects logistic regression | 17 110 observations  (51) | OR 2.15  (1.36–3.40) | n.t. | H 4 |
| *Subsample of the Nurses' Health Study II* (USA)  Women working as nurses in 1989. | | | | | | | | | | | | | |
| 29 | Lawn et al, 2022 | Exposure assessed 2008, follow-up with biannual surveys 2008-2015. | 53 (5) | Lifetime sexual harassment at work; one item, specifying physical and verbal harassment; No trauma / other (non-sexual harassment) trauma / sexual harassment | 12% | Incidence hypertension; self-reported high blood pressure; diagnosis or treatment. | 21% | Individuals with hypertension before 2008 excluded. Adjusted for race and ethnicity, parental education, somatotype at age 5, maternal and paternal hypertension, body mass index, alcohol use, smoking status, physical activity, diet quality 2007-2015, PTSD, Depression 2008. | Cox proportional hazards models | 33 127 (0) | HR 1.12  (1.03‒1.22) | ♀♀ | M 7 |

^a^ If nothing else is stated, mean age and (standard deviation). If only a percentage for age groups was reported, this is presented. Where the exact number for the analytical sample was not retrievable (not reported and not delivered on request), percentages as reported for the whole study sample are presented if available. This is indicated by the symbol ≈.

^b^ Instruments for exposure assessment: ISH=Inventory of Sexual Harassment, SEQ=Sexual harassment questionnaire, BSH=The Bergen Sexual Harassment Scale, VHA screen: Veterans Health Administration screening instrument, NAQ=Negative Acts Questionnaire.

^c^ Mean (standard deviation) for continuous measures, percent for categorical measures.

^d^ Instruments for outcome assessment: MDI = Major Depressive Inventory; CESD= Center for Epidemiologic Studies Depression Scale; BJSQ= Brief Job Stress Questionnaire; RAPI = Rutgers Alcohol Problems Index, ICD = International Statistical Classification of Diseases and Related Health Problems; EPDS = The Edinburgh Postnatal Depression Scale; GWS= General Well-being Scale of the Current Health Insurance Study Mental Health Battery; HSCL = Hopkins Symptoms Checklist.

^e^ Number of individuals in the analytical sample. Where the exact number for the analytical sample was not retrievable (not reported and not delivered on request), percentages as reported for the whole study sample are presented if available. This is indicated by the symbol ≈.

^f^ If nothing else is stated, numbers in brackets are the 95% confidence interval; SE = standard error, OR = odds ratio, HR = hazard ratio, IRR = incidence rate ratio, RR =risk ratio, ATET =average treatment effect of the treated.

^g^ Difference in the association between the exposure and outcome among women versus among men; n.t. = not tested; ♀=♂ = no gender difference found; ♀+ = stronger association in women ♂+ = stronger association in men; ♀♀ = only women in the sample; ♂♂ = only men in the sample.

^h^ Quality assessment and total score; M = Medium quality (6-9); H = High quality (0-5).
